# Therapeutic potential of *Laurus nobilis* extract by experimental and computational approaches: phenolic content and bioactivities for antioxidant, antidiabetic, and anticholinergic properties

**DOI:** 10.3389/fchem.2025.1541250

**Published:** 2025-02-19

**Authors:** Sevgi Altın, Mesut Işık, Cemalettin Alp, Emrah Dikici, Ekrem Köksal, Kevser Kübra Kırboğa, Mithun Rudrapal, Gourav Rakshit, Şükrü Beydemir, Johra Khan

**Affiliations:** ^1^ Department of Chemistry, Erzincan Binali Yıldırım University, Faculty of Science and Art, Erzincan, Türkiye; ^2^ Department of Bioengineering, Faculty of Engineering, Bilecik Şeyh Edebali University, Bilecik, Türkiye; ^3^ Science and Technology Application and Research Center, Aksaray University, Aksaray, Türkiye; ^4^ Department of Pharmaceutical Sciences, School of Biotechnology and Pharmaceutical Sciences, Vignan’s Foundation for Science, Technology and Research, Guntur, India; ^5^ Department of Pharmaceutical Sciences and Technology, Birla Institute of Technology, Ranchi, India; ^6^ Department of Biochemistry, Faculty of Pharmacy, Anadolu University, Eskişehir, Türkiye; ^7^ Department of Medical Laboratory Sciences, College of Applied Medical Laboratory Sciences, Majmaah University, Al Majma’ah, Saudi Arabia; ^8^ Health and Basic Science Research Center, Majmaah University, Al Majma’ah, Saudi Arabia

**Keywords:** *Laurus nobilis* L., phenolic content, antioxidant, antidiabetic, molecular dynamics, molecular docking

## Abstract

**Introduction:**

*Laurus nobilis* (LN), has traditional medicinal uses, and this study investigates its therapeutic potential by focusing on its phenolic content and bioactivities such as antioxidant, antidiabetic, and anticholinergic properties. Phenolic compounds play key roles in reducing oxidative stress and modulating enzymatic activities, relevant to metabolic and neurodegenerative disorders.

**Methods:**

LN leaf extracts were prepared via ethanol maceration, followed by filtration and concentration. Phenolic content was analyzed using LC-MS/MS. Antioxidant activity was assessed through ferric thiocyanate, DPPH, ABTS, and FRAP assays. Enzyme inhibition assays targeted AChE, BChE, and α-GLY, with IC50 values from dose-response curves. *In silico* analyses were conducted using molecular docking techniques to predict the binding mechanisms of identified phenolic compounds with the active sites of target enzymes, evaluating binding affinities and interaction profiles.

**Results:**

Vanillic acid and catechin hydrate were the most abundant phenolics. LN extract showed strong lipid peroxidation inhibition (50.53%) compared to Trolox (28.33%) and α-tocopherol (37.79%). Moderate radical scavenging and metal reduction potentials were observed. IC50 values were 2.57 µg/L for AChE, 3.78 µg/L for BChE, and 4.65 µg/L for α-GLY, indicating notable bioactivity. *In silico* studies confirmed strong binding affinities of phenolics to target enzymes.

**Discussion:**

LN extracts demonstrated promising antioxidant, antidiabetic, and anticholinergic activities, attributed to high phenolic content. Enzyme inhibition results suggest potential in managing metabolic and neurodegenerative disorders. *In silico* findings support these bioactivities, highlighting LN’s therapeutic potential.

## 1 Introduction

Many medicinal plants containing aromatic compounds are used as natural therapeutic agents and attract attention due to their rich bioactive properties (Veeresham, 2012; Zeng et al., 2020; Shi et al., 2022). Knowing the medicinal properties of plants on earth is essential due to their contributions to human health and the countries’ economies (Wangchuk and Tobgay, 2015; Sofowora et al., 2013). Studies reporting the biological activities of the plant extracts serve as a reference for discovering plants with therapeutic potential for many diseases (Altın et al., 2017; Balunas and Kinghorn, 2005; Tohma et al., 2019; Koksal, 2011). Plant extracts are the precursors of pure compounds responsible for their various biological activities, creating remarkable value for further research (Tonisi et al., 2020; Li et al., 2024). Traditionally used plants for therapeutic purposes can be analysed using modern techniques and may lead to obtaining novel promising compounds for treating some severe diseases (Dzobo, 2022).

Turkey has a rich flora and a long history of using plants for medicinal purposes. Turkey hosts many different plants because of their other geographical regions (Avci, 1996; Sargin and Büyükcengiz, 2019; Akan and Çakır, 2023). Turkey has over ten thousand plant taxa, and about four thousand are endemic (Avci, 1996; Sekercioglu et al., 2011). Some of these plants have been used for centuries by local people to treat various ailments, such as inflammation, infection, pain, diabetes, and neurological disorders. However, the scientific evidence for the efficacy and safety of these plants still needs to be improved. Therefore, there is a need for more comprehensive and systematic studies to evaluate the phytochemical and pharmacological properties of these plants and to identify their active constituents and mechanisms of action. *Lauraceae* is a plant family spreading in Turkey with a high economic and medicinal value. Only one species in Turkey represents *Lauraceae*, Laurus nobulis. *Laurus Nobilis* (LN) is a species native to the Mediterranean region, cultivated in many countries with temperate and subtropical climates. The commercial value of this species lies in its essential oils (Turgut et al., 2023). The leaves and fruit parts contain plenty of oil, and soap and skin cream are made using this oil. The leaves of the plant are dried and used as a spice. LN is a versatile and valuable plant with a long history of culinary and medicinal use.

LN has been reported to have various biological activities, such as antibacterial, antifungal, antiviral, antidiabetic, antispasmodic, anti-inflammatory, and antioxidant effects (Brinza et al., 2021). These activities are mainly attributed to the phenolic compounds present in the plant, especially flavonoids and phenolic acids. Phenolic compounds are secondary metabolites found naturally in the structure of plants and are delivered to humans and animals through the consumption of plant nutrients. These structures are an integral part of human and animal diets. Phenolic compounds form a significant group of natural antioxidants. For this reason, it is essential to determine which phenolic compounds are present in the structure of the plants whose antioxidant activity is investigated and what their amounts are. For this purpose, many chromatographic methods have been developed and used (Shi et al., 2022).

Phenolic compounds and flavonoids are secondary metabolites naturally found in plants, exhibiting a wide range of biological activities such as antioxidant, antimicrobial, neuroprotective, and anticancer effects (Sun and Shahrajabian, 2023). Flavonoid isomers such as Catechin, Myricetin, Naringenin, Luteolin, and Kaempferol also play crucial roles in modulating various biological pathways (Ysrafil et al., 2023). For instance, Catechin’s significant free radical scavenging capacity makes it a key candidate in mitigating oxidative stress-related diseases (Sheng et al., 2023). Myricetin has demonstrated neuroprotective properties, improving synaptic plasticity and showing promise for managing neurodegenerative conditions such as Alzheimer’s disease (Semwal et al., 2016; Ramezani et al., 2016; Wang et al., 2025; Tang et al., 2023). Similarly, Luteolin exhibits anti-inflammatory effects by inhibiting key enzymes like COX(Cyclooxygenase) and LOX (Chen et al., 2014; Dong et al., 2023), while Kaempferol (Kmp) has been shown to regulate apoptotic pathways, providing anticarcinogenic benefits (Qattan et al., 2022). Naringenin, with its antioxidant and antimicrobial properties, holds potential for addressing metabolic disorders (Cai et al., 2023). Among the isomers of these compounds, isocoumarins stand out for their potential to influence multiple biological processes, particularly in inflammation, cancer, and neurological disorders. Isocoumarins have been reported to inhibit key enzymes involved in inflammation, such as 5-lipoxygenase (5-LOX) and prostaglandin E2 (PGE2) synthase, thereby exerting anti-inflammatory effects. For instance, 3-aryl isocoumarin derivative 1c has been identified as a dual inhibitor effectively suppressing both 5-LOX and PGE2 production. This property suggests that isocoumarins may offer effects comparable to clinically utilized dual inhibitors like Licofelone (Ramanan et al., 2016). Moreover, isocoumarins demonstrate not only anti-inflammatory but also neuroprotective properties. A recent study revealed that an 8-hydroxy-3-aryl isocoumarin derivative exhibited neurotrophic effects by binding to the TrkB receptor and enhancing synaptic plasticity. This isocoumarin derivative increased dendritic arborization and microtubule-associated protein 2 (MAP2) expression in neuronal cells, shedding light on the mechanisms underlying its neuroprotective effects (Sudarshan et al., 2019). The TrkB receptor-mediated effects emphasize the therapeutic potential of isocoumarins in addressing central nervous system disorders such as Alzheimer’s disease and major depression. In this context, a comprehensive evaluation of the biological activities of phenolic compounds and flavonoids is crucial for the development of therapeutic approaches. Comparing derivatives like isocoumarins with traditional phenolic compounds offers a significant opportunity to deepen our understanding of their biological and pharmacological potentials. These studies aim to elucidate the interactions of phenolic compounds with therapeutically targetable enzymes and receptors, ultimately enhancing their applicability in biomedical fields.

Flavonoids are widely distributed throughout almost all parts of plants, including leaves, flowers, fruits, stems, and roots, and they represent the most abundant and structurally diverse subgroup of phenolic compounds (Panche et al., 2016; Roy et al., 2022). These secondary metabolites are particularly concentrated in the photosynthetic tissues of plants, such as leaves and flower petals, where they play critical roles in protecting plants against UV radiation and oxidative stress (Chen et al., 2022; Singh et al., 2023). Additionally, flavonoids contribute significantly to the vivid pigmentation of flowering plants, serving as essential natural colorants that attract pollinators and aid in reproductive processes (Elessawy et al., 2023). Their multifunctional presence underscores their importance in plant physiology and their potential therapeutic and industrial applications. Like many phenolic compounds, they cannot be synthesised by human and animal cells and must be obtained by consuming plant foods. Many studies have reported that plants’ therapeutic potential is due to their phenolic compounds, especially flavonoids (Tungmunnithum et al., 2018; Sun and Shahrajabian, 2023). Nearly 10,000 phenolic compounds have been detected in different plant sources, and about half of them are formed by flavonoids.

In addition to their antioxidant activity, phenolic compounds have been shown to have anticholinergic and antidiabetic effects. Anticholinergic agents inhibit the action of the neurotransmitter acetylcholine in the central and peripheral nervous system. They treat various conditions, such as Parkinson’s disease, Alzheimer’s disease, motion sickness, and overactive bladder (Durmaz et al., 2022; Bingol et al., 2021). Antidiabetic agents are substances that lower blood glucose levels and improve glucose metabolism. They treat diabetes mellitus, a chronic metabolic disorder characterised by hyperglycemia and impaired insulin secretion or action (Chaudhury et al., 2017). Phenolic compounds can modulate the activity of enzymes involved in the synthesis and degradation of acetylcholine, such as acetylcholinesterase and choline acetyltransferase, thus affecting the cholinergic system (Colović et al., 2013). Phenolic compounds can also modulate the activity of enzymes involved in glucose metabolism, such as alpha-glucosidase, alpha-amylase, and glucose-6-phosphatase, thus affecting glycemic control (Sarkar et al., 2021).

This study investigated the phenolic content and antioxidant, anticholinergic and antidiabetic potential of LN leaves. The phenolic compounds were identified and quantified by liquid chromatography-tandem mass spectrometry (LC-MS/MS). The antioxidant activity was evaluated using different methods, such as lipid peroxidation inhibition, radical scavenging (DPPH and ABTS), and metal reduction potentials. The anticholinergic activity was assessed by measuring the inhibition of AChE and BChE enzymes. The antidiabetic activity was evaluated by measuring the inhibition of alpha-glucosidase enzymes. The study’s results may support the traditional use of LN as a natural source of antioxidants and a potential remedy for neurological and metabolic disorders.

## 2 Materials and methods

### 2.1 Chemicals

2,2-diphenyl-1-picrylhydrazyl, gallic acid, Folin and Ciocalteu’s phenol reagent, quercetin (Cheng et al., 2024), ethylenediaminetetraacetic acid (EDTA), trihydroxymethylaminomethane (Tris), sodium citrate, 5,5′-dithio-bis (2-nitrobenzoic acid) (DTNB) which used in the studies were obtained from Sigma Aldrich.

### 2.2 Plant materials

LN L. leaves were collected from the vicinity of Esenpınar village, 750 m, Erdemli district of Mersin, Türkiye, during the plant’s flowering period in August 2021. Dr Ali Kandemir taxonomically defined the samples collected for analysis, and the herbarium samples were stored in Erzincan Binali Yıldırım University Herbarium with the code Kandemir 6,069.

### 2.3 Preparation of the extract

The collected plant material was dried at room temperature, in a place without ventilation problems and away from sunlight, and then crushed with liquid nitrogen to powder. The resulting sample was mixed with ethanol (1:10 ratio). Extraction was carried out in a shaker for 24 h. The solid was filtered through filter paper (Whatman No. 1) and evaporated. The dry extract was stored at +4°C until used in analysis studies.

### 2.4 LC-MS/MS analysis

The phenolic content of L. nobilis leaves was determined using LC–MS/MS, an analytical chemistry technique combining high-pressure liquid chromatography (UHPLC) and mass spectrometry (MS). This technique physically separates the compounds in the mixture by liquid chromatography, while mass spectrometry provides the structural identity and quantification of individual compounds with high sensitivity and specificity. A verification method was developed for 20 phenolic substances. Determining the phenolic compound in LN was done using the technique developed by Yilmaz (2020). In this method, the C18 Inertsil ODS-4 (3.0 mm × 100 mm, 2 μM) analytical column was used for chromatographic separation of analytes. This column is designed for reverse-phase chromatography and separates non-polar compounds. The column temperature was fixed at 40°C. This temperature is optimised to shorten the passage time of analytes through the column and increase solubility. The liquid chromatography system consists of components such as a SIL-30AC automatic sampling device, LC-30AD double pumps, CTO-10ASVP column oven and DGU-20A3R degasser. The elution gradient was established using mobile phase A (water and 0.1% formic acid) and mobile phase B (methanol and 0.1% formic acid). Formic acid was added to increase the ionisation of analytes and prevent corrosion in the ion source. The ratio of mobile phases was varied to improve the analytes’ escape times and peak shapes from the column. The sample injection volume was set as 4 μL, and the flow rate was kept at 0.5 mL min^-1^. Calculations were made with Lab Solutions software (Shimadzu, Kyoto, Japan) (Division shimadzu corporation, 2024). Analyses were measured using multiple reaction monitoring (MRM) mode. In this mode, the mass spectrometer records only the signals of the targeted compounds by selecting the analytes’ molecular ions and their fragmentation products (Yilmaz, 2020; Al-Khayri et al., 2022; Güven et al., 2023). In this way, background noise is reduced, and specificity is increased. In the study, three applications were made to determine each compound’s quantitative amount, and the results were averaged.

### 2.5 Antioxidant activity

#### 2.5.1 Inhibition of linoleic acid peroxidation

The assessment of the inhibitory effect of the LN ethanol extract on linoleic acid peroxidation was conducted using the ferric thiocyanate method, as outlined in the reference (Kavaz et al., 2021; Mitsuda et al., 1966). This methodology is based on quantifying the hydroperoxide generated during linoleic acid oxidation via spectrophotometric analysis at a wavelength of 500 nm. Elevated absorbance values indicate an excessive accumulation of peroxides resulting from the peroxidation process. These hydroperoxides subsequently catalyse the conversion of Fe2+ to Fe3+. Later, Fe3+ forms a coordination complex with the introduced thiocyanate reagent, which exhibits a peak absorbance at 500 nm.

#### 2.5.2 Radical scavenging activity

In this method, 2,2-diphenyl-1-picrylhydrazyl (DPPH•) scavenging activity of the plant extract was performed according to the method reported by Blois with slight modifications (Blois, 1958). Briefly, 0.26 mM solution of DPPH in 1 mL methanol was added to 3 mL of the sample solution in various concentrations. After mixing by vortex, the mixture was incubated for 30 min in the dark and at room temperature. The absorbance of the mixture was measured at 517 nm. Oxidants oxidise ABTS to the intensely coloured radical cation ABTS.+, and the antioxidant potential is determined as the ability of the test compounds to reduce colour by reacting directly with the ABTS radical (Re et al., 1999). ABTS (2 mmol L−1) solution was mixed with 2.45 mmol L−1 potassium persulfate (K2S2O8) solution. The new solution was incubated in the dark for 14 h at 25 °C. Firstly, ABTS.+ radical solution was diluted with sodium phosphate buffer (0.1 mol L−1, pH 7.4) until an absorbance of 0.750 ± 0.025 at 734 nm was obtained. The absorbance was measured using a spectrophotometer at 734 nm. The results were reported as per cent of radical scavenging activity.

#### 2.5.3 Iron (III) ion reducing capacity (FRAP)

The iron-reducing power capacity of the plant extract was determined according to the method reported by Oyaizu with slight modifications (Güder and Korkmaz, 2012). Accordingly, the stock solutions (1 mg/mL) of the extracts and standards were prepared in the test tubes containing 1.25 mL phosphate buffer (0.2 M, pH 6.6). Then, 1.25 mL of potassium ferric cyanide [K3Fe(CN)6] (1%) was added to the mixture and incubated at 50°C for 20 min. After 1.25 mL of 10% trichloroacetic acid (TCA) and 0.25 mL of 0.1% FeCl3 solution were added, the absorbances of the final mixture were measured spectrophotometrically at 700 nm. The results were expressed as mg TE/g extract.

#### 2.5.4 Cupric ions (Cu2+) (II) reducing capacity (CUPRAC)

This method was first discovered by Apak et al. (2006) and is a widely used effective method for determining antioxidant activity (Apak et al., 2013). It is based on reducing Cu2+ to Cu+ in the presence of neocuproine (2,9-dimethyl-1,10-phenanthroline). Cu + - neocuproine complex yields maximum absorbance at 450 nm. 1 mL of CuCl2 (0.01 M), 1 mL of neocuprin, and 1 mL of ammonium acetate (NH4Ac) as buffer solution were added to the test tube and mixed. Then, the different amounts of extract (10, 20, 40 μg mL−1) were added, and the total volume was adjusted to 4 mL with pure water. The absorbance was measured at 450 nm. The results were compared to standard antioxidants.

#### 2.5.5 Determination of total phenolic content

The extract’s total phenolic content was determined by the Folin-Ciocalteu method (Öztürk et al., 2022). Briefly, 100 μL of the stock solutions of the samples (1 mg/mL) were taken into test tubes containing 4.5 mL of distilled water. Then, 100 μL of Folin-Ciocalteu reagent and 300 μL of 2% Na2CO3 solution were added to the mixture. The mixture was vortexed and incubated for 120 min at room temperature. The absorbance of the mix was measured spectrophotometrically at 760 nm. A calibration curve was created with different concentrations of gallic acid used as a standard, and the phenolic content of the extracts was stated as mg gallic acid equivalent/g extract.

#### 2.5.6 Determination of total flavonoid content

The total flavonoid content of the plant extract was determined using the Aluminium chloride colourimetric method (Shraim et al., 2021). Accordingly, 100 μL of the extracts and standard stock solutions (1 mg/mL) was taken, and the volume was completed to 4.8 mL with methanol. Then, 100 µL of 1 M NH4CH3COO solution and 100 µL of 10% AlCl3 solution were added to the test tubes, and the mixture was incubated for 45 min at room temperature. After incubation, the absorbance of the mixture was measured spectrophotometerically at 415 nm. A calibration curve was created with different concentrations of quercetin used as a standard, and the flavonoid content of the extracts was given as mg quercetin equivalent/g extract.

#### 2.5.7 Cholinesterase inhibition assay

The inhibitory effect of the plant ethanol extract on cholinesterase (AChE and BChE) enzymes was determined using the Ellman spectrophotometric method described in reference (Ellman et al., 1961; Türkeş et al., 2022). A reaction solution containing 50 µL of 5,5′-dithio-bis(2-nitrobenzoic) acid (DTNB), 100 µL of Tris-HCl buffer (1 M, pH 8.0), and 50 µL of cholinesterase (5.32 × 10^−3^ U) was incubated at 30°C and mixed for 15 min. Subsequently, the reaction was initiated by adding 50 µL of acetylthiocholine iodide for AChE and butyrylthiocholine iodide for BChE as substrates. The enzymatic hydrolysis of the substrates was detected by spectrophotometry at 412 nm. The impact of the ethanol extract on cholinesterases was assessed across different concentration ranges. The IC50 values for the extract were calculated from activity (%)-[Inhibitor] graphs.

#### 2.5.8 α-GLY inhibition assay

The inhibitory potential of plant ethanol extract on α-GLY was assessed by employing p-nitrophenyl-D-glycopyranoside (p-NPG) as the substrate, following the established protocols in prior research endeavours (Türkeş et al., 2022; Tao et al., 2013). The absorbance values were quantified through spectrophotometric measurements at a wavelength of 405 nm. The impact of the ethanol extract on cholinesterases was assessed across different concentration ranges. The IC50 values for the extract were calculated from activity (%)-[Inhibitor] graphs.

### 2.6 In-silico studies

#### 2.6.1 Hardware and software utilised

Molecular docking studies were performed on a workstation with the following specifications: Operating System - Ubuntu 22.04 (LTS), 64-bit; Processor - Intel^®^ Core™ i5-12400 CPU @ 2.30 GHz; RAM - 16 GB; Graphics - 8 GB Nvidia GeForce RTX 3050 GPU. The protein structures in complexes with ligands were sourced from the Protein Data Bank (PDB) (Bernstein et al., 1978). Glide modules of Schrödinger software v2021.4 (Institute license: BIT Mesra) were employed for molecular docking studies (Friesner et al., 2006).

#### 2.6.2 Protein and ligand structure preparation for molecular docking

**FIGURE 1:**
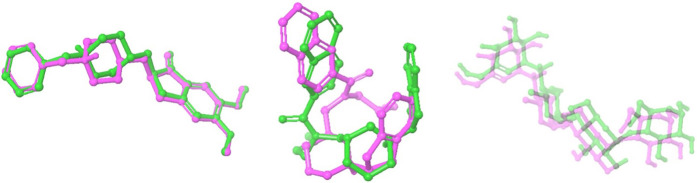
Overlay of docked ligand and crystallized conformation. This figure presents a superimposed view of the docked internal ligand (displayed in green) against its crystallised conformation (shown in pink), derived from the co-crystallized complex. The overlay provides a visual comparison of the ligand positions within the binding site, emphasising the accuracy of the docking process in replicating the actual ligand orientation observed in the crystal structure.

This study utilized three proteins, Recombinant Human Acetylcholinesterase in Complex with Donepezil (PDB ID:4EY7, 2.35 Å) (Cheung et al., 2012; Kang et al., 2018), Human Butyrylcholinesterase in Complex with 3F9 (PDB ID:4TPK, 2.70 Å) (Brus et al., 2014; Gao et al., 2019; Lu et al., 2020), and Human Lysosomal Acid-Alpha-Glucosidase, GAA, in Complex with acarbose (PDB ID:5NN8,2.45 Å) (Roig-Zamboni et al., 2017). The X-ray crystal structures of these proteins, each bound to an inhibitor, were obtained from the Protein Data Bank (PDB). The PDB structures utilized in this study were carefully selected based on multiple criteria to ensure their suitability for the docking studies (Figure 1). First, the chosen proteins are directly relevant to the study’s therapeutic focus, targeting acetylcholinesterase, butyrylcholinesterase, and lysosomal acid-alpha-glucosidase, all of which play crucial roles in the pathophysiology of neurodegenerative and metabolic disorders. Second, these structures (Table 1) were specifically selected because they are available in complex with known inhibitors, providing validated binding poses that serve as benchmarks for evaluating the binding potential of the tested compounds. This pre-complexed state ensures that the selected conformations are biologically relevant and optimized for inhibitor interactions. Third, the resolutions of the selected structures—2.35 Å (4EY7), 2.70 Å (4TPK), and 2.45 Å (5NN8)—were key factors in their selection. These high-resolution structures provide detailed atomic information, enabling precise modeling of protein-ligand interactions and minimizing inaccuracies in docking predictions.

**TABLE 1:** Bioactive ligands utilised in this study, along with their code, structure, and details.

Code	Compound	Structure	Details
MG1	Quercetin	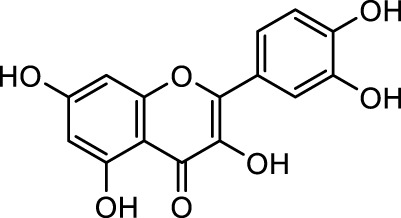	IUPAC Benzene carboxylic acid Chemical Formula: C7H6O2 Molecular Weight: 122.123 g/mol
MG2	Acetohydroxamic Acid	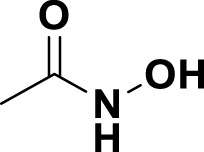	IUPAC N-Hydroxyacetamide Chemical Formula: C2H5NO2 Molecular Weight: 75.067 g/mol
MG3	Catechin hydrate	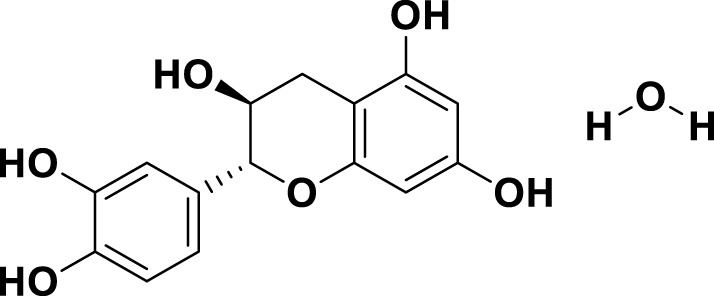	IUPAC: (2R,3S)-2-(3,4-dihydroxyphenyl)-3,4-dihydro-2H-chromene-3,5,7-triol; hydrate Chemical Formula: C15H16O7 Molecular Weight: 308.28 g/mol
MG4	Vanillic Acid	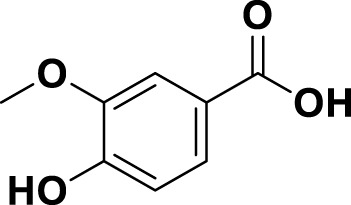	IUPAC 4-Hydroxy-3-methoxybenzoic acid Chemical Formula: C8H8O4 Molecular Weight: 168.148 g/mol
MG5	Resveratrol	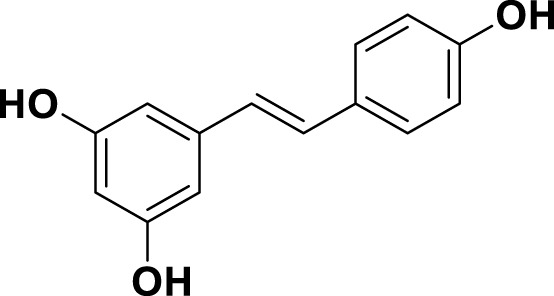	IUPAC 5-[(E)-2-(4-Hydroxyphenyl)ethen-1-yl]benzene-1,3-diol Chemical Formula: C14H12O3 Molecular Weight: 228.247 g/mol
MG6	Fumaric Acid	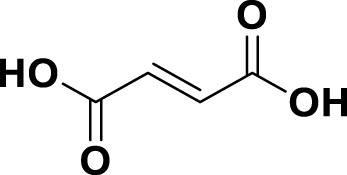	IUPAC: (2E)-But-2-enedioic acid Chemical Formula: C4H4O4 Molecular Weight: 116.072 g/mol
MG7	Gallic acid	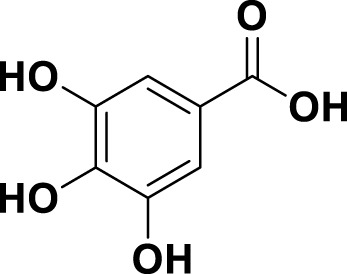	IUPAC 3,4,5-Trihydroxybenzoic acid Chemical Formula: C7H6O5 Molecular Weight: 170.12 g/mol
MG8	Caffeic Acid	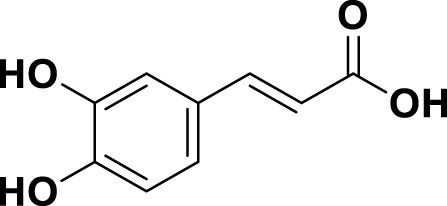	IUPAC 3-(3,4-Dihydroxyphenyl)-2-propenoic acid Chemical Formula: C9H8O4 Molecular Weight: 180.16 g/mol
MG9	Phloridzin dihydrate	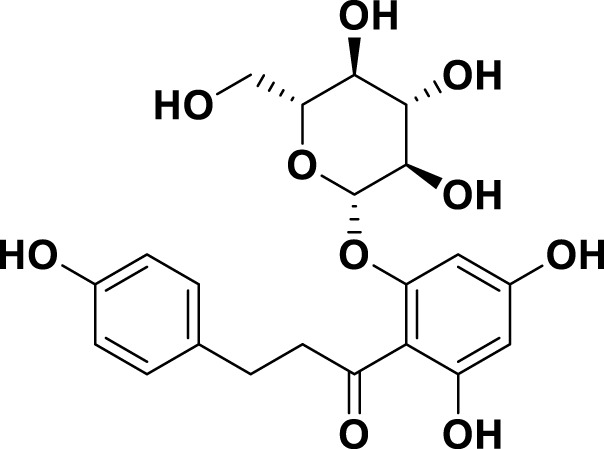	IUPAC: (2E)-But-2-enedioic acid Chemical Formula: C21H24O10 Molecular Weight: 436.413 g/mol
MG10	Oleuropein	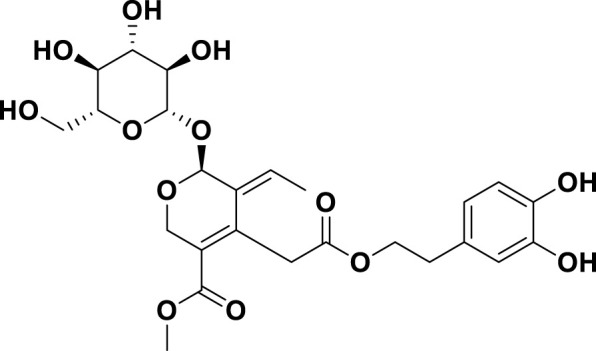	IUPAC: methyl (4S,5E,6S)-4-[2-[2-(3,4-dihydroxyphenyl)ethoxy]-2-oxoethyl]-5-ethylidene-6-[(2S,3R,4S,5S,6R)-3,4,5-trihydroxy-6-(hydroxymethyl)oxan-2-yl]oxy-4H-pyran-3-carboxylate Chemical Formula: C25H32O13 Molecular Weight: 540.5 g/mol
MG11	Ellagic Acid	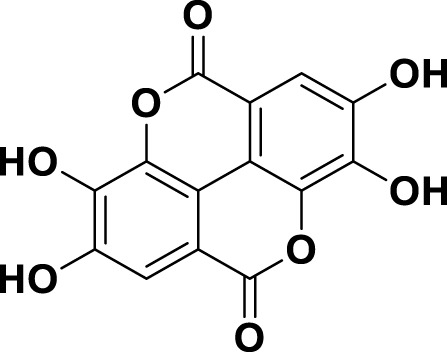	IUPAC 2,3,7,8-Tetrahydroxy[1]benzopyrano[5,4,3-cde][1]benzopyran-5,10-dione Chemical Formula: C14H6O8 Molecular Weight: 302.197 g/mol
MG12	Myricetin	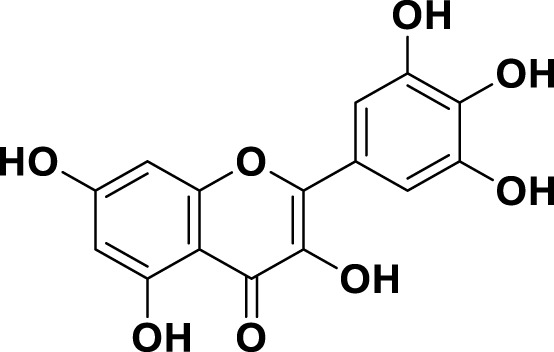	IUPAC 3,3′,4′,5,5′,7-Hexahydroxyflavone Chemical Formula: C15H10O8 Molecular Weight: 318.237 g/mol
MG13	Protocatechuic acid	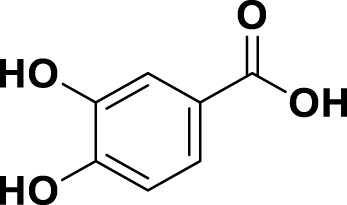	IUPAC 3,4-Dihydroxybenzoic acid Chemical Formula: C7H6O4 Molecular Weight: 154.12 g/mol
MG14	Butein	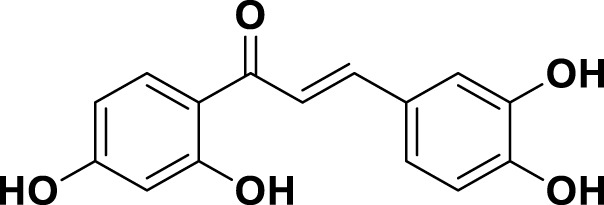	IUPAC 2′,3,4,4′-Tetrahydroxychalcone Chemical Formula: C15H12O5 Molecular Weight: 272.25 g/mol
MG15	Naringenin	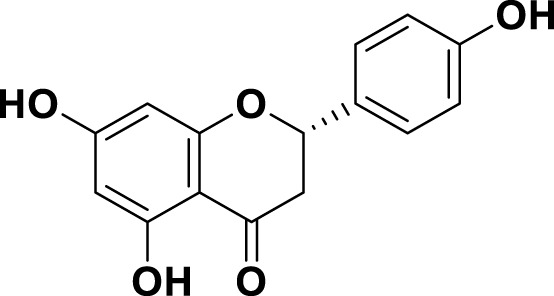	IUPAC: (2S)-4′,5,7-Trihydroxyflavan-4-one Chemical Formula: C_15_H_12_O_5_ Molecular Weight: 272.256 g/mol
MG16	Luteolin	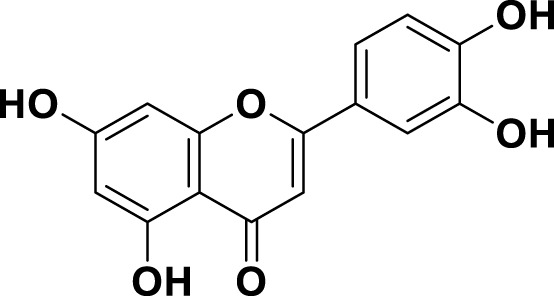	IUPAC 3′,4′,5,7-Tetrahydroxyflavone Chemical Formula: C_15_H_10_O_6_ Molecular Weight: 286.239 g/mol
MG17	Kaempferol	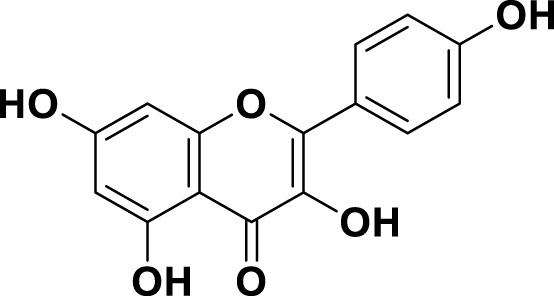	IUPAC 3,4′,5,7-Tetrahydroxyflavone Chemical Formula: C_15_H_10_O_6_ Molecular Weight: 286.23 g/mol
MG18	Alizarin	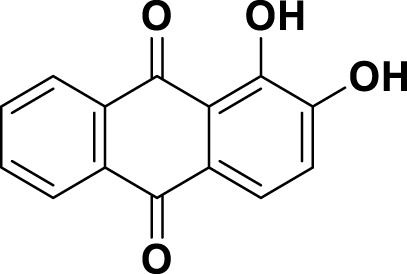	IUPAC 1,2-Dihydroxyanthracene-9,10-dione Chemical Formula: C_14_H_8_O_4_ Molecular Weight: 240.214 g/mol
MG19	4-Hydroxybenzoic Acid	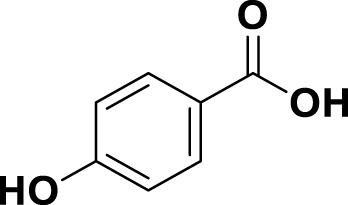	IUPAC: (1E,6E)-1,7-bis(4-hydroxy-3-methoxyphenyl)hepta-1,6-diene-3,5-dione Chemical Formula: C_21_H_20_O_6_ Molecular Weight: 368.39
MG20	Salicylic acid	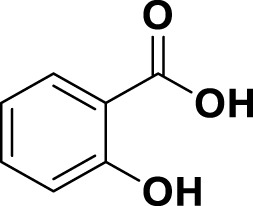	IUPAC 2-Hydroxybenzoic acid Chemical Formula: C_7_H_6_O_3_ Molecular Weight: 138.122 g/mol

Protein preparation was conducted using the protein preparation wizard module of Schrödinger software. This process included the addition of missing polar hydrogens, removal of water molecules beyond 5Å from hetero groups, ionization, generation of tautomeric states at pH 7.0 ± 2.0, optimization of hydrogen bonds, and energy minimization. The triple inhibitory potential of twenty phytochemicals or inhibitors was evaluated against these three proteins. Ligand preparation involved sketching the structures in ChemDraw, verifying their correctness with the structure checker module, generating 3D structures using the Chem3D module, and optimizing them with the LigPrep module using the OPLS force field X (Schrödinger Release 2021-3). During this process, compound tautomers were generated, maintaining the desired chirality for each compound.

#### 2.6.3 Validation of the docking program

Validation of a docking program is a critical step in assessing its reliability and accuracy in predicting ligand-protein interactions and evaluating the program’s ability to reposition native ligands into their crystallographically observed binding sites correctly. Successful redocking indicates that the program can reproduce known binding modes (Rakshit and Jayaprakash, 2023; Yele et al., 2022). This was performed for all three co-crystallized protein-ligand complexes.

#### 2.6.4 Protein-ligand docking

All molecular docking simulation studies were conducted using the ligand docking program within the Maestro 2021.4 module of Schrödinger (Friesner et al., 2006; Hu et al., 2024; Gao et al., 2023). Before initiating the docking process, the active site coordinates on the target proteins were generated using the Receptor Grid Generation module. The van der Waals radii scaling factor and partial charge cutoff were set at 1.0 and 0.25, respectively, while the remaining parameters were kept as default. The Extra Precision (XP) mode was employed to carry out molecular docking of the three phytocompounds in the active site of their respective proteins (Friesner et al., 2006; Sanapalli et al., 2022). Subsequently, the binding affinities and molecular interactions were analysed, and the data were recorded. Finally, the interactions within the protein-ligand complexes were visualised in 2D/3D using the ligand interaction module, and high-quality images were saved for representation.

#### 2.6.5 ADME and toxicity prediction

ADME (absorption, distribution, metabolism, and excretion) plays a pivotal role in predicting the pharmacodynamics of the molecule under study, potentially serving as a lead candidate for future drug development. The online web server SWISSADME, developed and maintained by the Swiss Institute of Bioinformatics (SIB) (https://www.swissadme.ch), was utilised for ADME evaluation based on molecular docking and dynamics results (Daina et al., 2017). The identified potential hit molecules were individually uploaded in SMILES format to the Marvin JS input panel on the website http://swissadme.ch/index.php. The server then performed *in silico* ADME predictions. In addition to ADME considerations, predicting toxicity is vital for assessing the safety profile of a drug. Using graph-based signatures, the pkCSM web server was employed to predict small-molecule pharmacokinetic properties (Pires et al., 2015). The web server database details toxicity, including AMES toxicity, maximum tolerated dose, hepatotoxicity, skin sensitisation, and hERG I and II inhibition. Toxicity assessments were conducted in prediction mode.

## 3 Results and discussion

### 3.1 The phenolic content of the LN

In this study, the phenolic content of the bay laurel plant was analysed using the LC-MS/MS method. Phenolic compounds are bioactive molecules that are essential to plants and human health. Human health has biological activities such as antioxidant, anti-inflammatory, anticancer, antidiabetic, antimicrobial, antiviral, antiallergic, antithrombotic and neuroprotective (Shahidi and Yeo, 2018; Rahman et al., 2021). The LC-MS/MS method is an effective method for identifying and quantifying these components. Thanks to this method, quality control of herbal products and supplements obtained from the bay plant can be made. Additionally, the potential health benefits of the phenolic components of the bay plant can be better understood. The LC-MS/MS method also provides information about the biosynthesis pathways and metabolic regulation of phenolic components of the bay plant (Mateos et al., 2020). This information is useful for understanding how environmental factors and genetic manipulation affect the bay plant’s production of bioactive compounds. Therefore, analysis of the phenolic content of the bay laurel plant by LC-MS/MS method is essential to advance the health effects of plant-based foods and drugs. Chromatographic and spectrometric parameters and the linear regression equations of standard phenolics and phenolic compounds in the LN samples are presented in Table 2, Supplementary Table S1 and Supplementary Figure S1. The phenolic content of the bay laurel plant was found by comparing it with standards. The most abundant compound was determined to be vanillic acid (4,599.00 μg/L). Vanillic acid was followed by catechin hydrate (3,351.53 μg/L). Therefore, these two compounds can be defined as the main compounds found in the structure of the LN. Luteolin (5.81 μg/L) and ellagic acid (5.47 μg/L) compounds were determined as the least common minor compounds in the structure of the LN. Vanillic acid has been identified as an essential secondary metabolite in many plant extracts whose biological activities have been investigated and isolated from many plants. Vanillic acid is a phenolic compound found in various dietary sources and herbs. In addition to being obtained from these biological sources, it is also synthesised chemically. It is used as a flavouring in different food products. It has been reported to have anticancer, antiobesity, antidiabetic, antibacterial, anti-inflammatory and antioxidant effects. In this study, it was reported for the first time that it was found to be the main compound in the bay laurel plant.

**TABLE 2:** Quantitative amounts of phenolic compounds in ethanol extracts of *Laurus nobilis*.

Standard compounds	^a^MRM	^b^LOD/LOQ (μg/L)	Recovery (%)	^c^RT	^d^R^2^	Concentration (µg/L)
Quercetin (MG1)	301.1 > 151	22.5/25.7	1.001	0.389	0.999	190,18
Acetohydroxamic Acid (MG2)	76.10 > 58.10	2.8/8.2	1.000	0.398	0.999	58,38
Catechin hydrate (MG3)	291.10 > 139.00	8.2/11.4	0.994	2,722	0.999	3351,53
Vanillic Acid (MG4)	168.80 > 93.00	125.5/142.2	1.001	2,885	0.998	4599,00
Resveratrol (MG5)	229.10 > 135.00	9.0/13.6	0.998	4,314	0.998	284,89
Fumaric Acid (MG6)	115.20 > 71.00	25.2/31.3	0.997	0.507	0.999	167,96
Gallic acid (MG7)	169.20 > 125.00	0.90/1.6	1.000	1.442	0.999	N.D.
Caffeic Acid (MG8)	179.20 > 135.00	6.3/10.7	1.009	2,778	0.996	17,25
Phloridzin dihydrate (MG9)	435.00 > 273.10	61.0/207.0	1.000	3,462	0.999	59,54
Oleuropein (MG10)	539.10 > 377.20	0.05/1.0	0.997	3.567	0.999	N.D.
Ellagic Acid (MG11)	300.90 > 145.10	0.101/0.333	1.002	3,900	1.000	5,47
Myricetin (MG12)	317.10 > 150.90	55.4/59.6	0.999	5.017	0.999	N.D.
Protocatechuic acid (MG13)	181.20 > 108.00	30.3/35.4	1.011	3.556	0.994	N.D.
Butein (MG14)	271.10 > 135.00	22.7/28.6	0.096	3,853	0.999	62,84
Naringenin (MG15)	271.10 > 150.90	5.4/6.4	0.998	3,879	0.996	N.D.
Luteolin (MG16)	285.20 > 132.90	0.5/2.5	1.007	4,124	0.998	5,81
Kaempferol (MG17)	285.10 > 116.90	206.6/214.3	0.999	4,115	0.999	68,46
Alizarin (MG18)	239.20 > 210.90	65.2/77.5	0.966	4.594	0.998	N.D.
4-Hydroxybenzoic Acid (MG19)	137.20 > 93.00	30.5/40.25	0.996	3.531	0.999	21,20
Salicylic acid (MG20)	137.20 > 93.00	4.2/7.6	1.009	3.534	0.999	27,44

^a^ MRM, multiple reaction monitoring.

^b^ LOD/LOQ (µg/L): limit of detection/limit of quantitation.

^c^ T, retention time.

^d^ R^2^: determination coefficient. N.D: not detected.

In 2021, Dobroslavić et al. (2021) evaluated the phenolic profile and antioxidant capacity of extracts obtained from bay leaves by green extraction techniques such as microwave-assisted extraction (MAE) and ultrasound-assisted extraction (UAE) with UPLC-MS/MS and ORAC methods. They detected 29 phenolic compounds in the extracts and found that kaempferol and quercetin glycosides were the most dominant among them. The extracts’ total phenolic content, flavonoid content, and antioxidant capacity were also determined. Green extraction techniques have advantages such as higher efficiency, shorter time, and less solvent and energy consumption than conventional extraction techniques. This study showed that bay leaves are a rich source of phenolic compounds, and green extraction techniques are suitable for obtaining these compounds. Dobroslavić et al., 29 phenolic compounds were detected in extracts obtained from LN L. Among these compounds, there are also compounds such as vanillic acid, catechin hydrate, luteolin and ellagic acid, which were also found in our study. This indicates that these compounds are essential among the phenolic components of the LN. According to Rúa et al. (2017), vanillic acid is a phenolic acid that has antioxidant, anti-inflammatory, antimicrobial, and anticancer activities. It is found in various plant sources, such as fruits, vegetables, herbs, spices, and tea. Catechin hydrate is a flavonoid with antioxidant, anti-inflammatory, antidiabetic, neuroprotective, and cardioprotective effects. It is widely distributed in many plant foods, especially tea, cocoa, grapes, and berries (Veiko et al., 2021). Luteolin is a flavone with antioxidant, anti-inflammatory, anticancer, antiallergic, and neuroprotective properties. It is present in many plant species, particularly in leaves, barks, clover blossoms, and herbs. Some dietary sources of luteolin include celery, broccoli, artichoke, parsley, thyme, and mint (Petkova et al., 2019). Ellagic acid is a polyphenol with antioxidant, anti-inflammatory, antimutagenic, and antitumor activities (Deepika and Maurya, 2022). It is produced in plants mainly by hydrolysis of ellagitannins, abundant in fruits, nuts, and bark. Some foods rich in ellagic acid include pomegranate, raspberry, strawberry, blackberry, walnut, and oak. These references show that these compounds are essential among the phenolic components of the *Laurus Nobilis*, as they have various biological activities and are widely distributed in plants. The LC-MS/MS method was used in both studies. This method is an effective method for the identification and quantification of phenolic compounds. However, the performance of the LC-MS/MS method depends on the chromatographic conditions, sample preparation methods, and standard compounds used. Therefore, the differences in these parameters used in both studies should be considered. In our analysis, the phenolic content of the bay laurel plant was found by comparing it with standards. The amounts of 6 of the 20 compounds used as standards could not be determined. This may mean these compounds are absent in the LN or below detectable levels. However, a more sensitive method or a more significant number of standard compounds may need to be used to determine whether these compounds are present in Bay Laurel. In addition, it would be useful to create a more comprehensive phenolic profile to detect other phenolic compounds in bay laurel.

Irakli et al. (2021) analysed the phenolic content of extracts obtained from solid wastes of plants belonging to the Lamiaceae family (thyme, sage, mint, basil, laurel) by LC-MS method. They detected 16 phenolic compounds in the extracts and reported that the Lamiaceae family also found rosmarinic acid, caffeic acid, chlorogenic acid, luteolin, and apigenin. In this study, evaluating solid wastes in terms of phenolic compounds is considered an environmentally and economically critical issue. In this study, it has been shown that the solid waste of the Lamiaceae family plant is a rich source of phenolic compounds and is a potential material for investigating the effects of these compounds on health. Our study analysed the phenolic content of extracts obtained from plant solid wastes using 100% ethanol solvent. This solvent was chosen to increase the solubility of phenolic compounds and reduce the effect of other compounds in the plant material. Our study shows that 100% ethanol solvent is similar to that of Irakli et al. (2021) and provides higher phenolic content, flavonoid content and antioxidant capacity than the 0.1% formic acid and acetonitrile mixture used by Irakli et al. (2021). These results reveal that the choice of solvent is an essential factor in the analysis of phenolic compounds and that ethanol is a suitable solvent for evaluating plant solid wastes.

LN has been extensively studied for its phenolic content and biological activities; however, comparisons with similar plants can significantly contribute to contextualizing the findings in such studies. In our research on LN, major phenolic compounds such as vanillic acid (4,599 μg/L) and catechin hydrate (3,351 μg/L) demonstrated superior lipid peroxidation inhibition compared to standard antioxidants such as Trolox (28.33%) and α-tocopherol (37.79%) with an inhibition rate of 50.53%. Similarly, studies on *Rosmarinus officinalis* (rosemary) have reported that phenolic compounds like rosmarinic acid and carnosic acid exhibit strong antioxidant activities when extracted using eco-friendly methods (Irakli et al., 2023). Likewise, species such as *Salvia ekimiana*, with phenolic profiles rich in vanillic acid, highlight the importance of this compound in antioxidant activities and its neurological effects (Orhan et al., 2012). Furthermore, *Origanum vulgare* (oregano), a rich source of vanillic acid, has demonstrated potent antioxidant effects, outperforming ascorbic acid and Trolox in reactive oxygen species (ROS) inhibition in H2O2-treated cells. Additionally, its antimelanogenic effects reveal a significant potential for biological activities (Chou et al., 2010).

The phenolic profile of LN is unique not only for its antioxidant activities but also for its DNA damage prevention properties. For instance, studies on the water extract of *Primula vulgaris* (WEP) have reported that phenolics such as p-coumaric acid and rutin are dominant, with a FRAP value of 82.63 ± 0.31 μM Trolox/g. Additionally, WEP has been shown to reduce H2O2-induced DNA damage in fibroblast cells in a concentration-dependent manner (Tugce Ozkan et al., 2017). When compared within this broader biological context, LN’s high vanillic acid content further underscores its distinctive potential. Finally, it has been reported that the co-processing of *Argania spinosa* oil and *Origanum vulgare* leaves enhances phenolic content and oxidative stability, demonstrating a synergistic approach that improves product quality and shelf life (Oubannin et al., 2024). While LN exhibits antidiabetic and anticholinergic effects that extend beyond its antioxidant capacity, comparative analyses with similar plants in terms of phenolic content and biological activities will contribute to the contextualization and standardization of these findings.

### 3.2 Antioxidant activity

The antioxidant activity of plants may vary depending on factors such as plant species, growing conditions, harvesting methods and extraction techniques (Tungmunnithum et al., 2018). Therefore, determining plants’ antioxidant activity is essential to understanding and evaluating their potential health and environmental benefits. In this study, we aimed to determine the antioxidant activity of LN L. leaves, which are widely consumed in the kitchen in many countries. In Table 3, the phenolic content of the plant extract was found to be 22.72 µg GAE/mg extract, and the flavonoid content was 57.36 µg QE/mg extract. These values indicate that the plant extract contains high amounts of phenolic and flavonoid compounds. DPPH and ABTS radical scavenging tests measure the plant extract’s ability to neutralise free radicals. In these tests, the radical scavenging activity of the plant extract was compared to the standard antioxidants BHA, BHT and Trolox. According to Table 3, the DPPH radical scavenging activity of the plant extract was found to be 43.47% and 48.75% for ABTS. These values indicate that the radical scavenging activity of the plant extract is lower than standard antioxidants (71.64%–82.95%).

**TABLE 3:** Radical removal and metal reduction activity *Laurus nobilis*.

Antioxidants	Total phenolic/flavonoid content	DPPH^a^ (0.3 mg mL^-1^)	ABTS^a^ (0.3 mg mL^-1^)	FRAP assay^b^ (0.2 mg mL^-1^)	CUPRAC assay^b^ (0.2 mg mL^-1^)
	(µg GAE/QE mg^-1^ extract)				
*Laurus nobilis*	22.72/57.36	43.47 ± 0.20	48.75 ± 1.20	0.249 ± 0.17	0.415 ± 0.03
BHA		71.64 ± 6.17	82.95 ± 6.37	0.43 ± 0.06	0.61 ± 0.04
BHT		46.67 ± 3.41	48.79 ± 3.20	0.65 ± 0.08	0.66 ± 0.05
Trolox		82.63 ± 6.37	79.68 ± 5.31	0.28 ± 0.01	0.52 ± 0.04

Standard antioxidants (BHA, butylated hydroxyanisole; BHT, butylated hydroxytoluene, trolox). GAE/QE: gallic acid equivalents/quercetin equivalent.

^a^ Values are expressed as per cent radical scavenging activity.

^b^ Values are expressed as absorbance. High absorbance indicates high metal reduction capacity.

**FIGURE 2:**
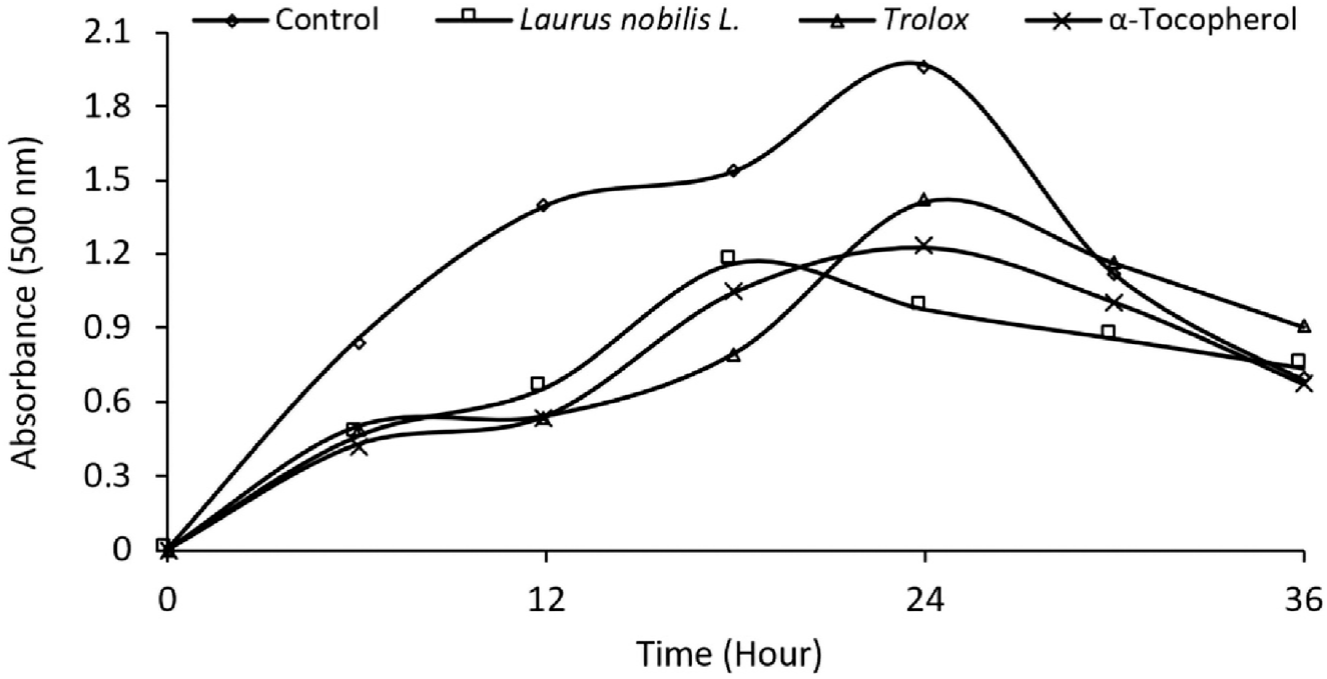
Comparative Inhibition of Linoleic Acid Peroxidation. This figure illustrates the inhibitory effects of standard antioxidants and ethanol extracts of *Laurus nobilis* L (20 μg/mL) on linoleic acid peroxidation. The data represent the extent of inhibition, highlighting the potential antioxidative properties of the extracts in comparison to established standards.

FRAP and CUPRAC metal reduction tests measure the ability of the plant extract to reduce metal ions. Metal ions can cause cell damage by increasing oxidative stress. Plant extract can reduce oxidative stress by reducing metal ions. In these tests, the metal-reducing activity of the plant extract was compared to the standard antioxidants BHA, BHT and Trolox. According to Table 3, the metal reduction activity of the plant extract in the FRAP test was 0.249, and the metal reduction activity in the CUPRAC test was 0.415. These values indicate that the metal-reducing activity of the plant extract is lower than the metal-reducing activity of standard antioxidants (0.43–0.66). In this way, determining the antioxidant activity of LN leaves is essential to reducing oxidative stress, revealing antifungal, antidiabetic, and anticholinergic effects, and evaluating the plant extract for different applications. Dhifi et al. (2018) DPPH, ABTS, FRAP and CUPRAC tests were used to measure the antioxidant activity of ethanol extract obtained from LN leaves. The IC50 values of ethanol extract were 0.3, 0.3, 0.2 and 0.2 mg/mL in DPPH, ABTS, FRAP and CUPRAC tests, respectively. This indicates that the ethanol extract poorly scavenges or reduces free radicals and metal ions. We can see that the antioxidant activity of LN in other regions and our region varies depending on the plant part, extraction solvent and method used. Essential oil from LN flowers appears to have the highest antioxidant activity in studies. Ethanol extract obtained from LN leaves has the lowest antioxidant activity. Table 3 shows that the radical scavenging and metal reduction activity of LN leaves is lower than that of standard antioxidants such as BHA, BHT, and Trolox. Therefore, the antioxidant activity of LN needs to be standardised, considering regional and methodological differences, to improve the quality and effectiveness of the plant extract and to determine the suitability of the plant extract for different applications. Figure 2 shows the effect of standard antioxidants and LN L. ethanol extracts (20 μg mL^-1^). Linoleic acid peroxidation is a measure of lipid oxidation. Lipid oxidation is a process that causes oxidative damage to cell membranes, proteins and DNA by producing free radicals (Ayala et al., 2014). Plant extracts can prevent lipid oxidation and reduce oxidative stress by inhibiting linoleic acid peroxidation (Dhifi et al., 2018; Awada et al., 2023). This may indicate the potential benefits of plant extracts to prevent or treat oxidative stress-related diseases. According to Figure 2, the extract inhibited lipid peroxidation by 50.53% after 24 h, compared to 28.33% and 37.79% by Trolox and α-tocopherol, respectively. The study suggests that LN L. ethanol extract inhibits linoleic acid peroxidation more effectively. The results indicate that LN L. ethanol extract may be a potential source of antioxidants to prevent lipid oxidation and reduce oxidative stress.

### 3.3 Antidiabetic and anticholinergic potential

In the community, the incidence of Type 2 diabetes has significantly increased, primarily attributed to ageing and overweight conditions. Inhibiting the activities of α-amylase and α-glucosidase enzymes can suppress carbohydrate digestion, delay glucose absorption, and reduce blood sugar levels. Slowing down glucose absorption provides additional time for insulin secretion by β-cells, ultimately enhancing the pharmacotherapeutic control of Type 2 diabetes. Therefore, exploring novel α-amylase and α-glucosidase inhibitors capable of decelerating or halting carbohydrate metabolism is critical in managing Type 2 diabetes (Atmaca, 2012). In this context, the ethanol extracts have shown promising results. The IC_50_ values of these extracts, indicating their inhibition effects on AChE and BChE as anticholinergic potential, were found to be 2.58 μg/L and 3.79 μg/L, respectively. Moreover, as an antidiabetic, the inhibition effect on α-GLY was found to be 4.65 μg/L (Table 4; Supplementary Figure S2). These findings suggest that these ethanol extracts could effectively inhibit α-amylase and α-glucosidase, thereby contributing to managing Type 2 diabetes. In individuals with diabetes, dietary therapy aims to reduce early postprandial hyperglycemia and control late postprandial hyperglycemia. Hence, medications that transiently inhibit the activities of enzymes in the gastrointestinal system are expected to mitigate postprandial glucose spikes effectively. Blocking α-amylase and α-glucosidase activities delays carbohydrate absorption, thereby preventing postprandial glucose elevation (Butterworth et al., 2011). Dirir et al. (2022) evaluated the α-amylase and α-glucosidase inhibition activities of 12 plant extracts. The results showed that plant extracts inhibited α-amylase activity by 10%–60% but inhibited α-glucosidase activity by 80%–100%. This study suggested that plant extracts may play a potential role in controlling postprandial hyperglycemia through α-glucosidase inhibition. Poovitha and Parani (2016) investigated the α-amylase and α-glucosidase inhibition activities and antioxidant capacities of 10 plant extracts. The results showed that plant extracts inhibited α-amylase activity by 20%–90% and α-glucosidase activity by 30%–100%. This study found a positive correlation between α-amylase and α-glucosidase inhibition and antioxidant capacity of plant extracts. Compared with our study, these studies show that the α-amylase and α-glucosidase inhibition activities of plant extracts vary depending on the plant, solvent, concentration and analysis method. Furthermore, these studies reveal that the α-glucosidase inhibition activity of plant extracts is higher than the α-amylase inhibition activity. This suggests that plant extracts inhibit the α-glucosidase enzyme more effectively to slow carbohydrate metabolism and prevent postprandial glucose elevation. It also shows that ethanol extracts may effectively inhibit Type 2 diabetes. Mssillou et al. (2020) investigated the chemical composition, antioxidant and antifungal effects of the essential oil obtained from LN flowers grown in Morocco. They showed that *L. nobilis* essential oil has a strong total antioxidant capacity (TAC) with the ability to scavenge free radical DPPH. This study shows that the essential oil obtained from L. nobilis flowers has significant antifungal and antioxidant activities due to its high level of 1,8-cineole.

**TABLE 4:** Inhibition effect of *Laurus nobilis* ethanol extract on AChE, BChE and α-glucosidase.

Inhibitors	AChE IC_50_ (µg/mL)	R^2^	BChE IC_50_ (µg/mL)	R^2^	α-glucosidase IC_50_ (µg/mL)	R^2^
*Laurus nobilis*	2.58	0.9911	3.79	0.994	4.65	0.9757
Tacrine	3.19	0.9896	2.54	0.9878		
Acarbose					3.15	0.9961

### 3.4 Molecular docking analysis

The present study employs molecular docking and ADME analyses to predict the interactions and pharmacokinetic properties of the compounds under investigation. While these computational methods provide valuable insights and are widely recognized as essential tools in drug discovery and development, it is important to acknowledge their inherent limitations. Computational predictions, although robust, may not always fully replicate the complexity of biological systems. Therefore, future studies should aim to incorporate experimental validation, such as *in vitro* and *in vivo* assays, to confirm the computational findings. This integration of experimental approaches would not only enhance the reliability of the results but also provide a more comprehensive understanding of the real-world interactions and therapeutic potentials of the compounds. Such a combined methodology would further strengthen the translational relevance of the findings and contribute to the development of more effective therapeutic agents.

#### 3.4.1 Validation of the docking program

The validation model of proteins with PDB ID: 4EY7, 4TPK, and 5NN8 revealed predicted binding energy of −16.9623, −13.6368, and −10.2322 kcal and reference RMSD of 0.815, 0.514, and 0.462 Å. The overlay confirmation of the internal ligands with their co-crystallized conformations has been shown in Figure 2.

#### 3.4.2 Docking

The docking analysis of all ligands with the three proteins indicated favourable binding energies and inhibition constants. Notably, it was observed that nearly all ligands exhibited a superior binding affinity compared to the reference internal ligand. This observation underscores the practical accommodation of the ligands within the sub-pockets of all three proteins. The binding energies, docking scores, and inhibition constants for all molecules are provided in Table 5.

**TABLE 5:** Results of molecular docking study of 20 natural product analogues against PDB ID: 4EY7, 4TPK, and 5NN8.

Ligand code	Binding affinity/Docking score of ligands (kcal/mol)					
	Protein ID: 4EY7		Protein ID: 4TPK		Protein ID: 5NN8	
	Glide score	Glide energy	Glide score	Glide energy	Glide score	Glide energy
MG1	−104.728	−445.388	−857.568	−470.811	−632.384	−354.436
MG2	−404.947	−206.707	−342.002	−171.309	−258.114	−177.402
MG3	−116.426	−411.835	−788.135	−432.203	−555.464	−343.572
MG4	−503.073	−227.416	−482.051	−225.903	−329.187	−175.679
MG5	−813.859	−349.491	−641.676	−391.736	−418.447	−334.843
MG6	−175.516	−135.348	−158.337	−106.483	−177.372	−620.704
MG7	−652.698	−248.471	−516.443	−228.568	−52.501	−139.267
MG8	−594.813	−236.833	−506.936	−21.822	−442.585	−225.287
MG9	−994.026	−554.522	−113.674	−575.109	−610.376	−475.614
MG10	−132.312	−647.063	−113.349	−65.368	−762.853	−472.404
MG11	−12.042	−431.189	−804.857	−403.251	−587.159	−35.096
MG12	−112.578	−465.925	−933.071	−38.201	−661.733	−376.156
MG13	−621.367	−217.483	−563.581	−174.145	−432.082	−195.925
MG14	−135.282	−471.769	−841.473	−406.698	−613.093	−327.361
MG15	−918.431	−416.205	−730.281	−433.583	−578.454	−322.395
MG16	−101.879	−458.971	−817.514	−466.215	−608.195	−333.539
MG17	−99.949	−397.095	−778.845	−432.854	−529.828	−33.405
MG18	−10.713	−389.422	−738.376	−368.297	−444.138	−334.634
MG19	−532.888	−195.572	−445.109	−189.536	−342.589	−145.945
MG20	−556.356	−19.779	−528.975	−18.397	−394.646	−153.281

Upon observation, it was noted that the top 25% of compounds identified exhibited the most negative binding energies. Specifically, Oleuropein and Myricetin were found to interact with all three proteins, demonstrating substantial binding potential. Table 6 shows the top 25% of ligands obtained through docking-based screening against all three proteins. These two molecules, Oleuropein and Myricetin, demonstrated strong binding with all three proteins. They effectively accommodated within the active site and interacted with the residues, forming hydrogen bonds, various hydrophobic interactions, and other molecular interactions. The effective binding energies can be attributed to numerous hydroxy functional groups in these ligands. These groups facilitate hydrogen bonding with various amino acid residues in the active site, contributing to the overall stability of the ligand-protein interactions.

**TABLE 6:** Tabular representation of the top 25% ligands obtained through docking-based screening against all three proteins.

Binding affinity/Docking score of ligands (kcal/mol)								
Ligand Code	Protein ID: 4EY7		Ligand	Protein ID: 4TPK		Ligand	Protein ID: 5NN8	
	Glide score	Glide energy	Code	Glide score	Glide energy	Code	Glide score	Glide energy
MG14	−135.282	−471.769	MG9	−113.674	−575.109	MG10	−762.853	−472.404
MG10	−132.312	−647.063	MG10	−113.349	−65.368	MG12	−661.733	−376.156
MG11	−12.042	−431.189	MG12	−933.071	−38.201	MG1	−632.384	−354.436
MG3	−116.426	−116.426	MG1	−857.568	−470.811	MG14	−613.093	−327.361
MG12	−112.578	−465.925	MG14	−841.473	−406.698	MG9	−610.376	−475.614

#### 3.4.3 Interaction analysis

We chose the top 5 ligands (25%) that exhibited strong binding with all three proteins. Upon critical observation, it was noted that the top-scoring compounds showed favourable docking scores, indicating that they retained all the structural requirements to be considered suitable ligands. Additionally, these compounds displayed promising pharmacophores. Among these top 5 hit molecules, Oleuropein and Myricetin demonstrated promising inhibitory results against all three selected proteins. Oleuropein and Myricetin displayed H-bonding with Tyr341, Thr75, Arg296, Phe295, Ser293 and Asp74 residues. Individually, Tyr341 (Tyrosine 341) acts as a key component in substrate binding and stabilisation, contributing to the formation of the acetylcholine binding pocket (Figure 2), Thr75 (Threonine 75) plays a role in stabilising the ligand through hydrogen bonding and contributes to the overall architecture of the active site, Arg296 (Arginine 296) functions in substrate recognition and binding, facilitating interactions with acetylcholine and other ligands, Phe295 (Phenylalanine 295) plays a role in forming hydrophobic interactions within the active site, contributing to substrate recognition and stabilisation, Ser293 (Serine 293) partakes in the hydrogen bonding network within the active site, influencing the stability of ligand binding, Asp74 (Aspartic acid 74) plays a crucial role in the catalytic mechanism of acetylcholinesterase, serving as a key residue involved in substrate hydrolysis. The interaction with Tyr337 is critical for inhibiting this human enzyme (Ashani et al., 1994). Hydrophobic residues, such as Phe297 and Phe338, form hydrophobic pockets within the active site. These interactions are crucial for stabilising ligands and substrates through nonpolar forces. They also aid in substrate specificity and are often involved in conformational changes of proteins. Hydrophobic residues may undergo dynamic rearrangements in the active site to accommodate different ligands or substrates. Aromatic residues, such as phenylalanine (Phe338) in acetylcholinesterase, can engage in π-π stacking with aromatic rings of ligands, suggesting a role in substrate recognition. These interactions also contribute to the overall stability of protein structures and can influence the catalytic mechanism of enzymes. However, the variations in the binding patterns of these flexible ligands, spanning the active site gorge across related species of acetylcholinesterase, are evident. The probable cause for such distinctions lies in the subtle alterations observed in the shape of the gorge during our analysis. It is anticipated that these differences in gorge shape may significantly impact the binding affinity of elongated inhibitors with conformational variability. This is particularly pertinent for dual acetylcholinesterase (AChE) inhibitors, characterised by moieties separated by a tether that simultaneously binds to both the peripheral and catalytic sites. The ligand interactions are detailed in Table 7 and Supplementary Figure S3.

**TABLE 7:** Tabular representation of the top 25% ligands obtained through docking-based screening against protein with PDB ID: 4EY7.

Sl. No.	Code	Docking interactions with active site amino acid residues	H-bond distance (Å)
1	MG14	H-bond- Tyr337, Asp74, Arg296, and Phe295 Hydrophobic- Phe338, His447, Trp86, Phe297, Tyr124, Val294, Ser293, Leu289, and Trp286 π-π stacking- Tyr341	1.33, 1.12, 1.21, and 2.81
2	MG10	H-bond- Tyr341, Thr75, Arg296, and Phe295 Hydrophobic- Ser203, Glu202, Trp86, Gly121, Gly122, Tyr124, Phe297, Val294, Ser293, Leu289, Trp286, Tyr72, Asp74, Leu76, Gly342, and Phe338 π-π stacking- Tyr337 and His447	2.87, 2.81, 2.33 and 2.23
3	MG11	H-bond- Ser293 and Phe295 Hydrophobic- Leu289, Phe338, Val294, Arg296, Phe297, Asp74 and Tyr72 π-π stacking- Tyr341, Trp286, and Tyr124	3.21 and 2.95
4	MG3	H-bond- Phe295 Hydrophobic- Ser293, Val394, Arg296, Phe297, His447, Tyr124, Gly121, Tyr337 and Tyr72 π-π stacking- Tyr341, Phe338, and Trp286	2.96
5	MG12	H-bond- Arg296, Ser293 and Asp74 Hydrophobic- Trp286, Leu289, Val294, Phe395, Phe297, Tyr72, Tyr124, Phe338 and Tyr337 π-π stacking- Tyr341	2.80, 3.08 and 3.20

#### 3.4.4 Ligand interaction with human butyrylcholinesterase (PDB ID: 4TPK)

Oleuropein and Myricetin displayed H-bonding with His438, Glu197, Gly115 and Ala328 residues. Individually, His438 (Histidine 438) has two roles; firstly, it is involved in the catalytic mechanism of butyrylcholinesterase, acting as a general base or acid during substrate hydrolysis. Secondly, it plays a crucial role in stabilizing the transition state of the enzymatic reaction, facilitating the efficient breakdown of substrates. Glu197 (Glutamic Acid 197) is crucial for butyrylcholinesterase’s catalytic activity. It may act as a general base or acid, aiding the hydrolysis of substrates and active site configuration. Glu197 contributes to shaping the active site and assisting in substrate recognition through its interactions with ligands. Gly115 (Glycine 115), a flexible structural element, introduces flexibility to the active site, which is essential for accommodating various ligands and substrates with different sizes and conformations. Gly115 may undergo conformational changes during substrate binding, contributing to the induced-fit mechanism of butyrylcholinesterase, Ala328 (Alanine 328), being an alanine residue, contributes to the hydrophobic environment within the active site. This is crucial for stabilising hydrophobic portions of substrates or inhibitors, helps maintain the structural integrity of the active site, and may participate in forming hydrophobic interactions with ligands. The hydrophobic residues collectively contribute to a protein’s structure, stability, and function by forming a hydrophobic core within the protein. Their interactions drive the formation of secondary and tertiary structures, determining the overall three-dimensional conformation of the protein. Hydrophobic residues can contribute to ligand recognition by forming interactions with hydrophobic regions of ligands. They may play a role in the conformational dynamics of proteins. Herein, the π-π stacking interactions majorly contribute to the adaptability of the active site to different ligands. The ligand interactions are detailed in Table 8 and Supplementary Figure S4.

**TABLE 8:** Tabular representation of the top 25% ligands obtained through docking-based screening against protein with PDB ID: 4TPK.

Sl. No.	Code	Docking interactions with active site amino acid residues	H-bond distance (Å)
1	MG9	H-bond- Ala328, Pro285, Ser287, His438 and Tyr128 Hydrophobic- Leu286, Val288, Gly117, Gly116, Gly115, Tyr114, Thr120, Phe398, Trp231, Gly121, Thr122, Gly122, Gly439, Ala199, Leu125, Ser198, Glu197, Trp82, Phe329 π-π stacking- Tyr332	1.58, 2.20, 2.83, 3.20, 2.21
2	MG10	H-bond- His438, Glu197 and Gly115 Hydrophobic- Trp231, Phe398, Gly121, Thr120, Gln119, Asp70, Gly439, Tyr440, Ile442, Ser198, Ala199, Gly117, Gly116, Tyr114, Tyr128, Trp82, Ser79, Tyr332, Pro285, Leu286, Ser287, Val288 and Ala328 π-π stacking-Phe329	3.82, 3.96, 1.55
3	MG12	H-bond- Glu197, Gly115 and Ala328 Hydrophobic- Thr120, Gly121, Gly116, Tyr114, Tyr128, Ile442, Ser198, Tyr440, Gly439, His438, Met437, Trp430, Gly78, Met434, Phe329, Val331, Tyr332 and Asp70 π-π stacking- Trp82	1.82, 1.56 and 1.23, 1.35
4	MG1	H-bond- Glu197 and Leu286 Hydrophobic- Pro285, Ser287, Val288, Phe398, Trp82, Gly439, Ile442, Tyr128, Ser198, Gly115, Gly116 and Gly117 π-π stacking- His438, Phe329 and Trp231	2.25, 3.02 and 2.85
5	MG14	H-bond- His438, Glu197 and Leu286 Hydrophobic- Ser287, Val288, Phe398, Ser198, Glu197, Tyr128, Ile442, Tyr440, Gly439, Trp82, Gly117, Gly116 and Gly115 π-π stacking- His438, Phe329 and Trp231	2.23, 1.85, 2.86 and 2.94

#### 3.4.5 Ligand interaction with human lysosomal acid-alpha-glucosidase (PDB ID: 5NN8)

The narrow substrate-binding pocket of this protein is located near the C-terminal ends of β-strands of the catalytic (β/α) domain and is shaped by a loop from the N-terminal β-sheet domain and both inserts I and II. Oleuropein and Myricetin displayed H-bonding with Asp616, Arg600, Asp282, Ser676, Asp404, and Asp518 residues. Hydrogen bonding with Asp616 may play a role in the enzyme’s catalytic mechanism, potentially stabilising intermediates during substrate hydrolysis. It also contributes to the architecture of the active site, influencing substrate binding and catalysis, Arg600 are likely involved in substrate recognition, contributing to the enzyme’s specificity for certain substrates. These interactions may help stabilise the substrate within the active site, facilitating the enzymatic reaction. Hydrogen bonds with Asp282 may be essential for the enzyme’s catalytic activity, potentially participating in proton transfer during substrate hydrolysis and contributing to maintaining the structural integrity of the active site. Ser676 likely contributes to the enzyme’s overall stability and conformational integrity and may play a role in the dynamic behaviour of the active site, influencing conformational changes during catalysis. Hydrogen bonds with Asp404 may be involved in the catalytic mechanism, potentially assisting in substrate binding and positioning for hydrolysis, shaping the active site and influencing the enzyme’s catalytic properties. Hydrogen bonds with Asp518 are likely crucial for substrate recognition and binding and contribute to the specific configuration of the active site, influencing the enzyme’s catalytic efficiency. The hydrophobic residues are likely key players in substrate recognition, catalysis, and the overall stability of the enzyme, making them potential targets for therapeutic interventions or modifications for specific functionalities. The ligand interactions are detailed in Table 9 and Supplementary Figure S5.

**TABLE 9:** Tabular representation of the top 25% ligands obtained through docking-based screening against protein with PDB ID: 5NN8.

Sl. No.	Code	Docking interactions with active site amino acid residues	H-bond distance (Å)
1	MG10	H-bond- Asp616, Arg600 and Asp282 Hydrophobic- Leu678, Leu677, Ser676, His674, Gly615, Trp613, Phe649, Leu650, Trp481, Arg672, Asp645, Trp516, Asp518, Met519, Asp404, Leu405, Ile441, Trp376, Ser523, Phe525, Ala555, Arg281, Leu283 and Ala284	2.80, 2.86, 2.83 and 3.20
2	MG12	H-bond- Ser676, Asp404 and Asp518 Hydrophobic- Asp616, Arg600, Trp613, Trp516, Ile441, Trp481, Trp376, Leu405, Leu650, Leu678 and Leu677 π-π stacking- Phe649	2.24, 2.35, 2.84
3	MG1	H-bond- Ser676 and Asp518 Hydrophobic- Met519, Asp616, Arg600, Trp481, Ile441, Leu405, Asp404, Trp376, Leu650, Gly651, Leu677 and Leu678 π-π stacking- Phe649	1.82, 1.56 and 1.23
4	MG14	H-bond- Ser676, Leu677, Asp518, Arg600 and Asp616 Hydrophobic- Trp618, Tyr292, Asp282, Met519, Trp481, Ile441, Leu650, Gly651, Ser679, Trp376 and Leu678 π-π stacking- Phe649	2.25, 2.50, 2.12, 2.35 and 2.45
5	MG9	H-bond- Arg411, Asp518, Asp404 and Asp616 Hydrophobic- Leu678, Leu677, Ser676, His674, Trp516, Ile441, Arg672, Arg600, Leu405, Trp613, Trp376, Gly615, Leu650, Tyr292 and Asp282 π-π stacking- Trp481 and Phe649	2.25, 2.40, 2.55 and 3.02

**FIGURE 3:**
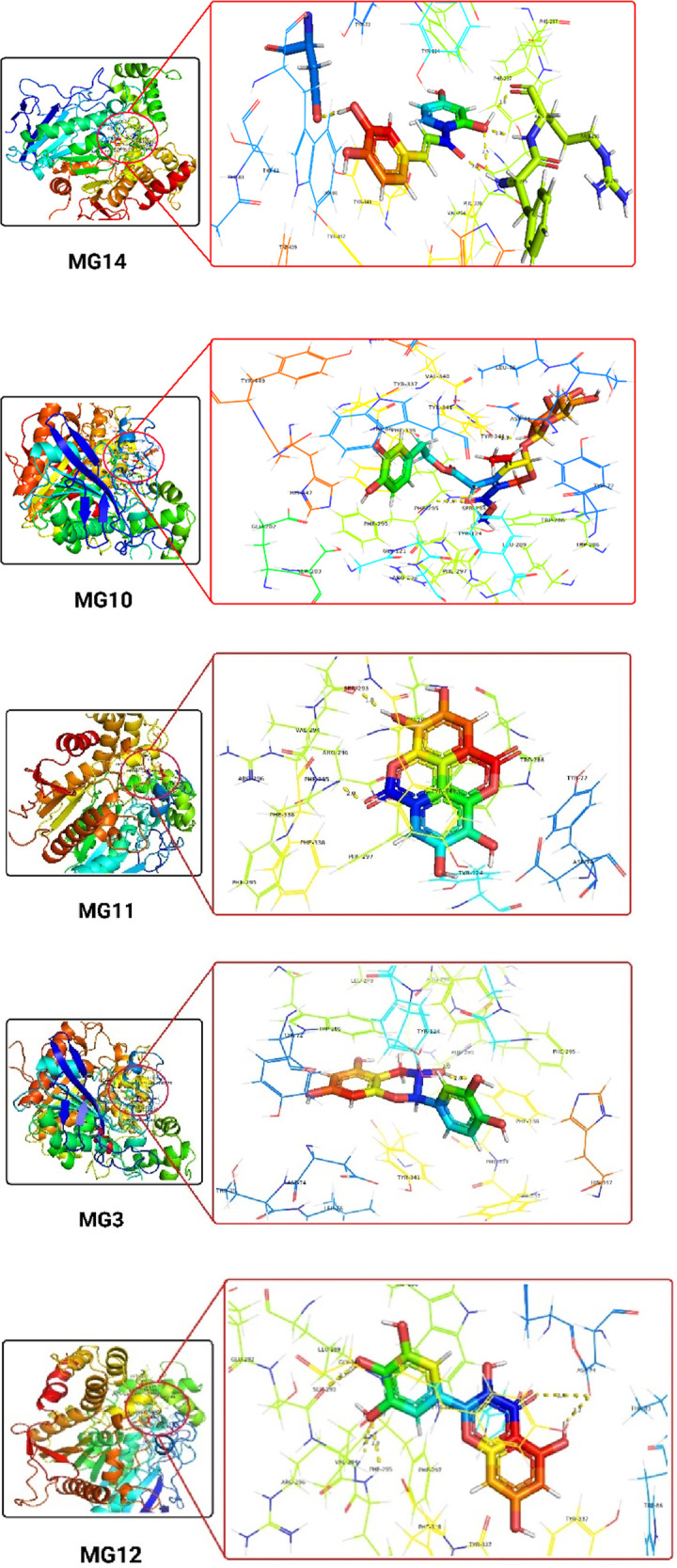
The detailed 3D atomic interactions of the top 5 ligands at the active site of recombinant human acetylcholinesterase (PDB ID: 4EY7) .

**FIGURE 4:**
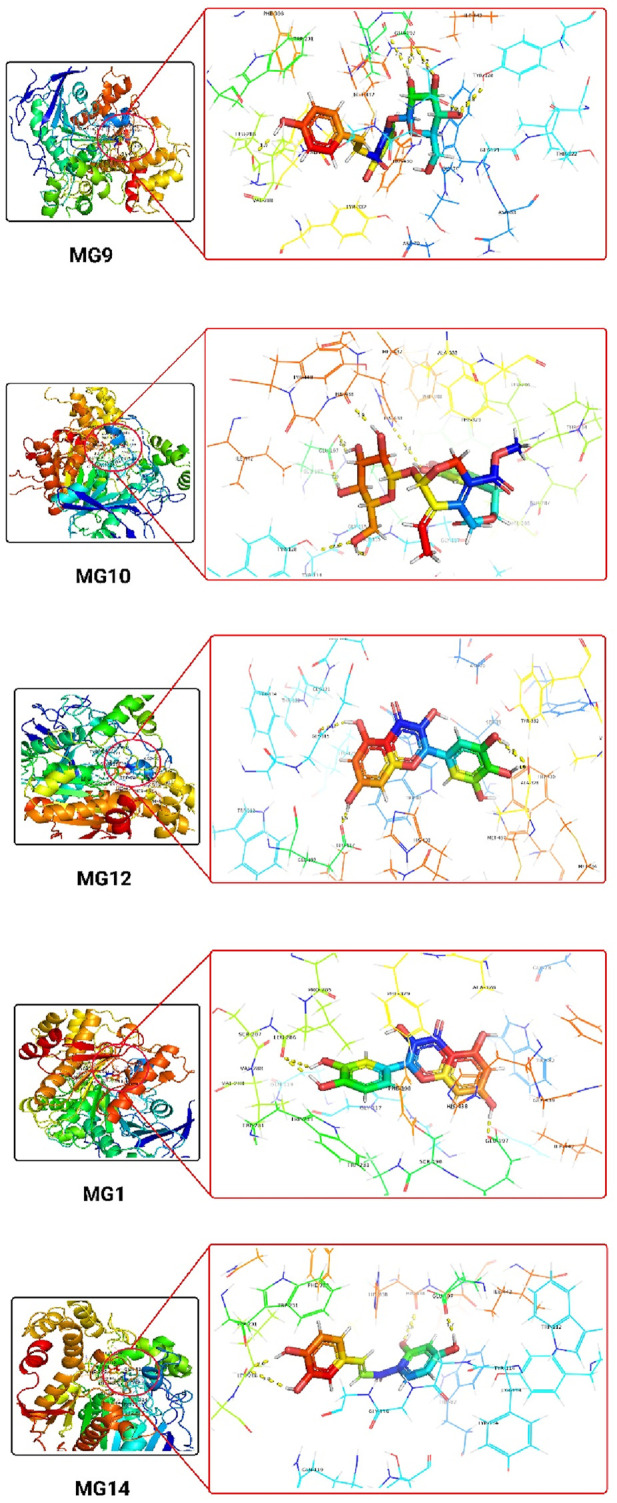
The detailed 3D atomic interactions of the top 5 ligands at the active site of recombinant human butyrylcholinesterase (PDB ID: 4TPK).

**FIGURE 5:**
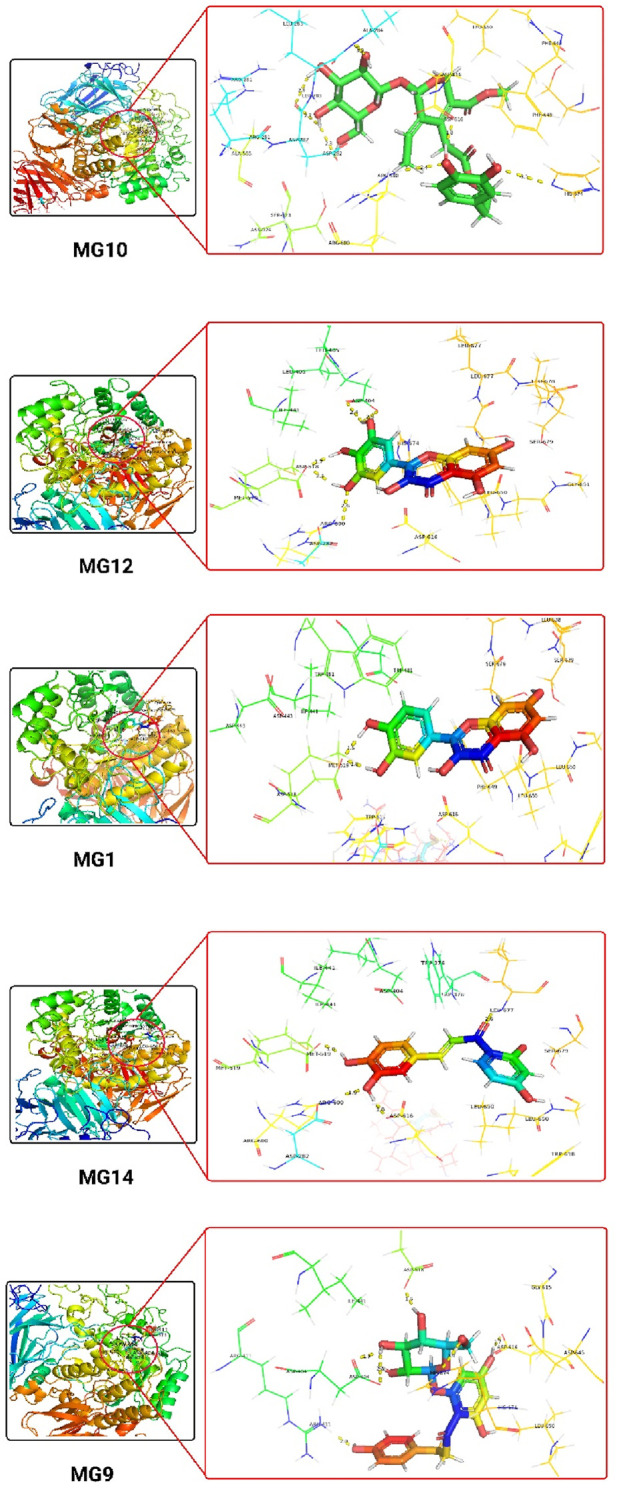
The detailed 3D atomic interactions of the top 5 ligands at the active site of Human lysosomal acid-α-GLY (PDB ID: 5NN8).

The 3D docking images of the best top scored compounds against 4EY7, 4TPK and 5NN8 are displayed in Figures 3–5, respectively.

#### 3.4.6 ADME analysis

The ADMET profiles of the two primary ligands investigated were assessed using online web servers, specifically SwissADME (http://www.swissadme.ch/). A comprehensive presentation of the acquired ADME properties is provided in Table 10. The fate of a compound within the human body is frequently assessed through its ADME properties, which encompass absorption, distribution, metabolism, and elimination. This evaluation sheds light on the molecule’s behaviour and interactions within the human physiological system (Guan et al., 2019; Ferreira and Andricopulo, 2019; Norinder and Bergström, 2006). The findings revealed that the molar refractivity, reflecting the overall polarity of the molecules, was 104.70 for Oleuropein and 92.42 for Myricetin. These values fell within the typical range (30–140) (Ertlet al., 2000). The topological polar surface area (TPSA) for Oleuropein and Myricetin was measured at 88.60 and 59.66 Å^2^, respectively. TPSA reflects a molecule’s potential for cell permeation, with values greater than 140 Å^2^ indicating poor permeability. Generally, a TPSA less than 90 Å^2^ is preferred for molecules to traverse the blood-brain barrier (BBB) and exert an effect on the central nervous system (CNS) (Ertl et al., 2000). This implies that both compounds can cross the blood-brain barrier (BBB). Regarding drug properties influencing ADMET, the “solubility class lipophilicity” pertains to a molecule’s capacity to dissolve in a lipophilic medium (Arnott and Planey, 2012). These properties encompass permeability, absorption, distribution, metabolism, excretion, solubility, plasma protein binding, and toxicity. Results of iLOGP (Daina, et al., 2014) and SILICOS-IT suggested that the iLOGP values of all the molecules were in the acceptable range (3.72 and 3.51), while SILICOS-IT values for all the identified leads were in the most favourable range (−4.40 and −4.01). Water solubility is crucial in influencing a drug’s distribution and absorption. Log S calculations provide insight into the molecule’s solubility in water at 25 °C. According to the ESOL model, calculated log S values should not exceed six for optimal solubility (Delaney, 2004). Oleuropein and Myricetin exhibited log S values of −3.92 and −3.50, indicating a favourable solubility profile. Based on these results, all lead compounds demonstrated a harmonious balance between permeability and solubility, suggesting potential acceptable bioavailability upon oral administration. Additionally, all molecules showed highly predicted gastrointestinal (GI) absorption (Daina and Zoete, 2016). Understanding the results of ADMET and cell-based bioassays is aided by permeability predictions (Potts and Guy, 1992). Results indicated that the permeability over human skin was −6.25 and −5.82 cm/s for Oleuropein and Myricetin. These predicted values were acceptable (Potts and Guy, 1992). Drug interactions resulting from metabolism can sometimes diminish a drug’s bioavailability. Drug-metabolizing enzymes can only interact with the drug in its unbound state. Cytochrome P450 enzymes (CYPs), the most significant class of metabolizing enzymes, must be investigated to comprehend the metabolic behaviour of our molecules. The inhibitory activity of all lead compounds on cytochrome P450 enzymes, specifically those in human liver microsomes (HLM), was assessed (Cortes and Vapnik, 1995). A molecule’s drug-likeness indicates its potential to be developed into an oral medication. In our study, drug-likeness was evaluated using five different filters. All molecules passed without violating drug-likeness rules and achieved a bioavailability score of 55% (indicating good bioavailability). The Abbott Bioavailability score, determined by feasibility scores of 11%, 17%, 56%, or 85%, predicts the fate of a chemical for an experiment, including quantifiable Caco-2 cell line permeability or 10% oral bioavailability (in rats) (Martin, 2005). PAINS and Brenk techniques were employed to uncover potential ambiguous regions that might lead to false-positive biological results (Brenk et al., 2008; Baell and Nissink, 2018). All molecules were found to comply with PAINS and Brenk rules. Additionally, the synthetic accessibility for all molecules was estimated (Ertl and Schuffenhauer, 2009). According to the criteria, all compounds exhibited a moderate level of hardness on a scale ranging from 1 (easy) to 10 (very tough). Consequently, based on the provided data, it is evident that the predicted ADME data for both molecules fall within the recommended values.

**TABLE 10:** Description of *in silico* ADME parameters of all three ligands under study.

	Compounds code		MG10	MG12
ADME PROFILE	Physiochemical parameters	Formula	C_25_H_32_O_8_	C_15_H_10_O_8_
		Molecular weight	540.518 g/mol	318.197 g/mol
		Mol. Refractivity	104.70	92.42
		TPSA	88.60 Å^2^	59.66 Å^2^
	Lipophilicity	ILOGP	3.72	3.51
		SILICOS-IT	−4.40	−4.01
	Water Solubility	Log S (ESOL), Class	−3.92 Soluble	−3.50 Soluble
	Pharmacokinetics	GI absorption	High	High
		Plasma Protein Binding (human)	70.35	79.22
		BBB permeant	No	Yes
		Log K_p_(skin perm.)	−6.25 cm/s	−5.82 cm/s
		CYP1A2	No	Yes
		CYP2D6	No	Yes
	Drug-likeness Rules	Lipinski (Pfizer)	Yes	Yes
		Ghose (Amgen)	Yes	Yes
		Veber (GSK)	Yes	Yes
		Egan (Pharmacia)	Yes	Yes
		Muege (Bayer)	Yes	Yes
		Bioavailability Score	0.55	0.55
	Medicinal Chemistry	PAINS	0 alert	0 alert
		Brenk	0 alert	0 alert
		Synthetic accessibility	2.92	2.37

#### 3.4.7 Toxicity

The toxicity profiles of the top two molecules (Oleuropein and Myricetin) were assessed using the online web server pkCSM (https://biosig.lab.uq.edu.au/pkcsm/prediction). The AMES test, which utilises microorganisms to predict the mutagenic potential of a chemical compound, yielded positive results for all molecules, indicating no AMES toxicity. The maximally tolerated dose (MTD), representing the highest dose most patients can take, was technically calculated. For Oleuropein and Myricetin, the maximum tolerated doses (human) were 0.486 and 0.722 Log mg/kg/day, respectively, indicating a moderate dosage level according to established protocols, with both being the most potent. hERG I and II (human Ether-a-go-go-Related gene) codes for proteins regulating ion channels crucial for the cardiac electrical action potential of the heart. Therefore, during drug development, drugs need to avoid inhibiting these channels. Only Myricetin showed no hERG I and II inhibition, minimising the likelihood of ventricular arrhythmias. The Oral Rat Acute Toxicity (LD50) values were 3.721 and 2.484 mol/kg, respectively, while the Oral Rat Chronic Toxicity (LOAEL) values were 4.484 and 3.307, respectively, indicating a favourable safety profile. All molecules were predicted to be non-hepatotoxic and demonstrated no skin sensitisation. The toxicity levels for T. Pyriformis and Minnow were within acceptable ranges. Furthermore, Myricetin’s non-inhibition of hERG I and II highlights its potential for enhanced cardiac safety, an important consideration during drug development. While these computational predictions provide promising insights, the importance of experimental validation is recognised. Future studies will aim to experimentally verify these predictions, including key parameters such as AMES test outcomes and hERG channel interactions, to strengthen the translational relevance of these findings. A detailed presentation of the expected toxicity results for all molecules under study is provided in Table 11.

**TABLE 11:** Tabular representation data of predicted toxicity identified leads.

Model name	Units	Compounds name	
		MG10	MG12
AMES toxicity	Yes/No	No	No
Max. Tolerated dose (human)	Log mg/kg/day	0.468	0.722
hERG I inhibitor	Yes/No	No	No
hERG II inhibitor	Yes/No	Yes	No
Oral Rat Acute Toxicity (LD50)	Mol/kg	3.721	2.484
Oral Rat Chronic Toxicity (LOAEL)	Log mg/kg_bw/day	4.484	3.307
Hepatotoxicity	Yes/No	No	No
Skin Sensitisation	Yes/No	No	No
T. Pyriformis toxicity	Log ug/L	0.285	0.285
Minnow toxicity	Log mM	4.093	1.402

The stability of bioactive compounds is a critical parameter for their pharmaceutical application. Although this study primarily focuses on the biological activities of the identified compounds, future research could incorporate a detailed stability assessment. Such studies could include evaluations under various conditions such as temperature, pH, and light exposure to determine the long-term viability of these compounds. Stability testing not only provides insights into the shelf-life of the compounds but also their feasibility for formulation into pharmaceutical products. Exploring stabilization strategies, such as encapsulation or the use of stabilizing agents, may further enhance the applicability of these compounds. This consideration would complement the current findings and expand their relevance in practical applications.

## 4 Conclusion

The LC-MS/MS analysis revealed the presence of various phenolic compounds in the LN, with the significant levels being vanillic acid and catechin hydrate. These compounds are known for their antioxidant properties and may contribute to the observed biological activities of the LN. In conclusion, this study sheds light on the antioxidant and anticholinergic potential of LN leaves and provides valuable information about the phenolic content in the LN. These findings may pave the way for further research into the therapeutic applications of this plant, especially in the context of neurodegenerative diseases such as Alzheimer’s. Moreover, observations show that the ligands, especially Oleuropein and Myricetin, exhibit strong binding affinities with effective interactions at the active site. The findings from this study add valuable information to the development of receptor-targeted ligand therapies (triple inhibitors). The study also highlights the importance of ADME assessment in predicting the pharmacokinetics of our best results. The use of tools such as SWISSADME and the pkCSM web server contributes to a comprehensive understanding of the drug-likeness and toxicity profiles of identified hit molecules. Further experimental validation and optimisation of these two hit molecules identified *in silico* are warranted for future drug development efforts.

## Data Availability

The original contributions presented in the study are included in the article/Supplementary Material, further inquiries can be directed to the corresponding authors.
