# Supplementary material for: Therapeutic potential of Laurus nobilis extract by experimental and computational approaches: phenolic content and bioactivities for antioxidant, antidiabetic, and anticholinergic properties
Source: Front Chem. 2025 Feb 19;13:1541250. doi: 10.3389/fchem.2025.1541250 (PMC11880275; doi:10.3389/fchem.2025.1541250)

***Supplementary Material***

**Table S1.** LC-MS/MS parameters and calibration equations for standard phenolic compounds.

| **Standard compounds** | **ESI ION**  **MOD** | **^a^MRM** | **^b^RSD %** | **^C^LOD/LOQ (μg/L)** | **Recovery (%)** | **^d^RT** | **Equation** |
| --- | --- | --- | --- | --- | --- | --- | --- |
| Quercetin | neg | 301.10>150.90 | 0.0136 | 22.5/25.7 | 1.001 | 3.891 | Y=(13.7831)X+(-146.951) |
| Acetohydroxamic acid | pos | 76.15>58.00 | 0.0082 | 2.8/8.2 | 1.000 | 0.406 | Y = (150.982)X + (23.1833) |
| Catechin hydrate | neg | 291.00>139.10 | 0.0236 | 8.2/11.4 | 0.994 | 2.532 | Y = (79.2933)X + (-2406.22) |
| Vanillic acid | pos | 168.95>65.00 | 0.0062 | 125.5/142.2 | 1.001 | 2.762 | Y = (48.0522)X + (-876.904) |
| Resveratrol | pos | 229.00>135.00 | 0.0131 | 9.0/13.6 | 0.998 | 3.606 | Y = (46.4361)X + (-1314.61) |
| Fumaric acid | neg | 115.20>71.10 | 0.0047 | 25.2/31.3 | 0.997 | 0.809 | Y = (20.2986)X + (-762.592) |
| Gallic acid | neg | 169.10>124.90 | 0.0136 | 0.90/1.6 | 1.000 | 1.278 | Y = (65.3835)X + (-2699.84) |
| Caffeic acid | neg | 179.00>135.00 | 0.0137 | 6.3/10.7 | 1.009 | 2.836 | Y = (124.785)X + (-487.132) |
| Phloridzin dihydrate | neg | 435.10>273.10 | 0.0564 | 61.0/207.0 | 1.000 | 3.594 | Y = (33.4069)X + (-1396.90) |
| Oleuropein | neg | 539.10>377.00 | 0.0694 | 0.05/1.0 | 0.997 | 3.567 | Y = (25.9240)X + (-558.916) |
| Ellagic acid | neg | 301.10>228.90 | 0.0856 | 0.101/0.333 | 1.002 | 3.681 | Y = (13.1516)X + (717.421) |
| Myricetin | neg | 317.00>179.10 | 0.0079 | 55.4/59.6 | 0.999 | 3.644 | Y = (5.25903)X + (-1167.31) |
| Protocatechuic acid | neg | 181.00>108.00 | 0.0129 | 30.3/35.4 | 1.011 | 3.556 | Y = (37.0934)X + (2684.23) |
| Butein | neg | 271.00>134.90 | 0.0145 | 22.7/28.6 | 0.096 | 3.935 | Y = (526.954)X + (23026.1) |
| Naringenin | neg | 271.00>150.90 | 0.0205 | 5.4/6.4 | 0.998 | 3.952 | Y = (49.3543)X + (367.917) |
| Luteolin | neg | 285.00>150.90 | 0.0057 | 0.5/2.5 | 1.007 | 4.069 | Y = (317.241)X + (33733.3) |
| Kaempferol | neg | 285.00>117.00 | 0.0144 | 206.6/214.3 | 0.999 | 4.298 | Y = (34.6668)X + (3721.79) |
| Alizarin | neg | 239.00>211.00 | 0.0351 | 65.2/77.5 | 0.966 | 4.594 | Y = (2.63905)X + (-206.494) |
| 4-Hydroxybenzoic acid | neg | 137.20>93.10 | 0.0154 | 30.5/40.25 | 0.996 | 3.555 | Y = (3.97487)X + (1614.23) |
| Salicylic acid | neg | 137.20>93.00 | 0.0124 | 4.2/7.6 | 1.009 | 3.558 | Y = (735.804)X + (-498.102) |

^a^MRM: Multiple Reaction Monitoring. ^b^RSD %: Relative standard deviation. ^c^LOD/LOQ (µg/L): Limit of detection/ limit of quantitation. ^d^RT: Retention time. ^e^R2: Determination coefficient. N.D: Not detected.

Figure S1. Representative LC-MS/MS chromatogram for standard phenolic compounds.

|  | **Name** |  |
| --- | --- | --- |
| 1 | Acetohydroxamic Acide_poz |  |
| 2 | Catechinhyrate_pos |  |
| 3 | Vanilic Acide_poz |  |
| 4 | Syringic acid_pos |  |
| 5 | Thymoquinone_poz |  |
| 6 | Resveratrol_pos |  |
| 7 | Fumaric Acid _neg |  |
| 8 | Gallic acid_neg |  |
| 9 | Caffeic acid_neg |  |
| 10 | Hydoxycinamic_neg* |  |
| 11 | 4-Hydroxybenzoic acid _neg |  |
| 12 | Protocatechuic acid _neg |  |
| 13 | Salicylic acid_neg |  |
| 14 | Oleuropein_neg |  |
| 15 | Phloridzindyhrate_neg |  |
| 16 | 2-hyroxy1.4nph_neg |  |
| 17 | Myricetin_neg |  |
| 18 | Ellagic acid_neg |  |
| 19 | Quercetin_neg |  |
| 20 | Bütein_neg |  |
| 21 | Naringenin_neg |  |
| 22 | Silymarin_neg |  |
| 23 | luteolin_neg |  |
| 24 | Kaempferol_neg |  |
| 25 | Alizarin_neg |  |
| 26 | Curmin_neg |  |
|  |  |  |

**Figure S2.** Percent inhibition effect of *Laurus nobilis* ethanol extract against AChE, BChE and α-glucosidase

**
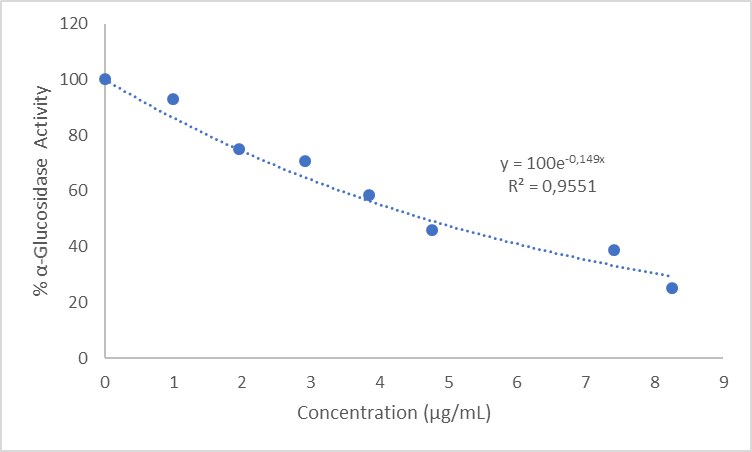

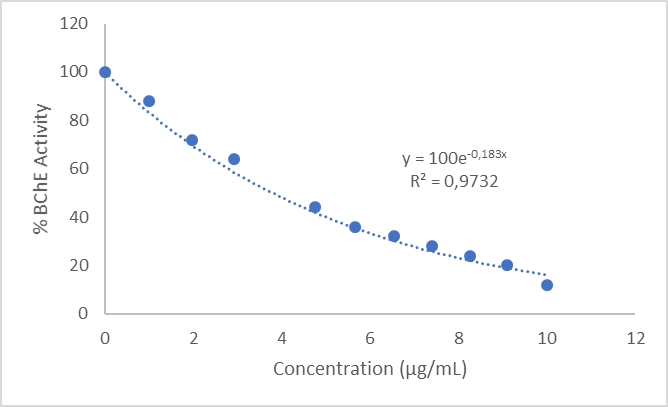

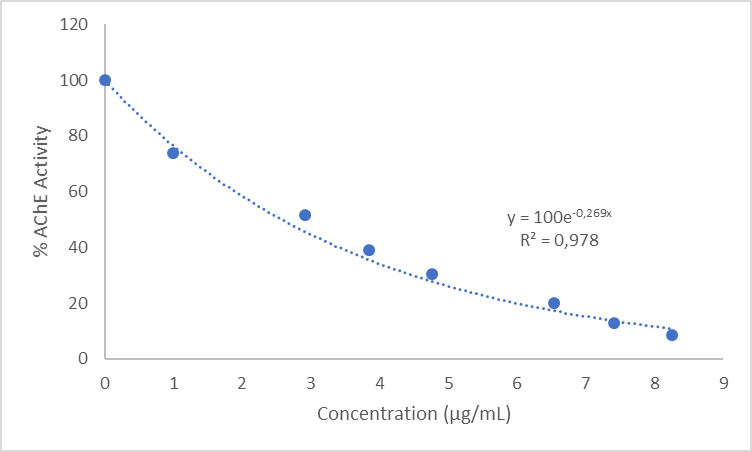
**

**
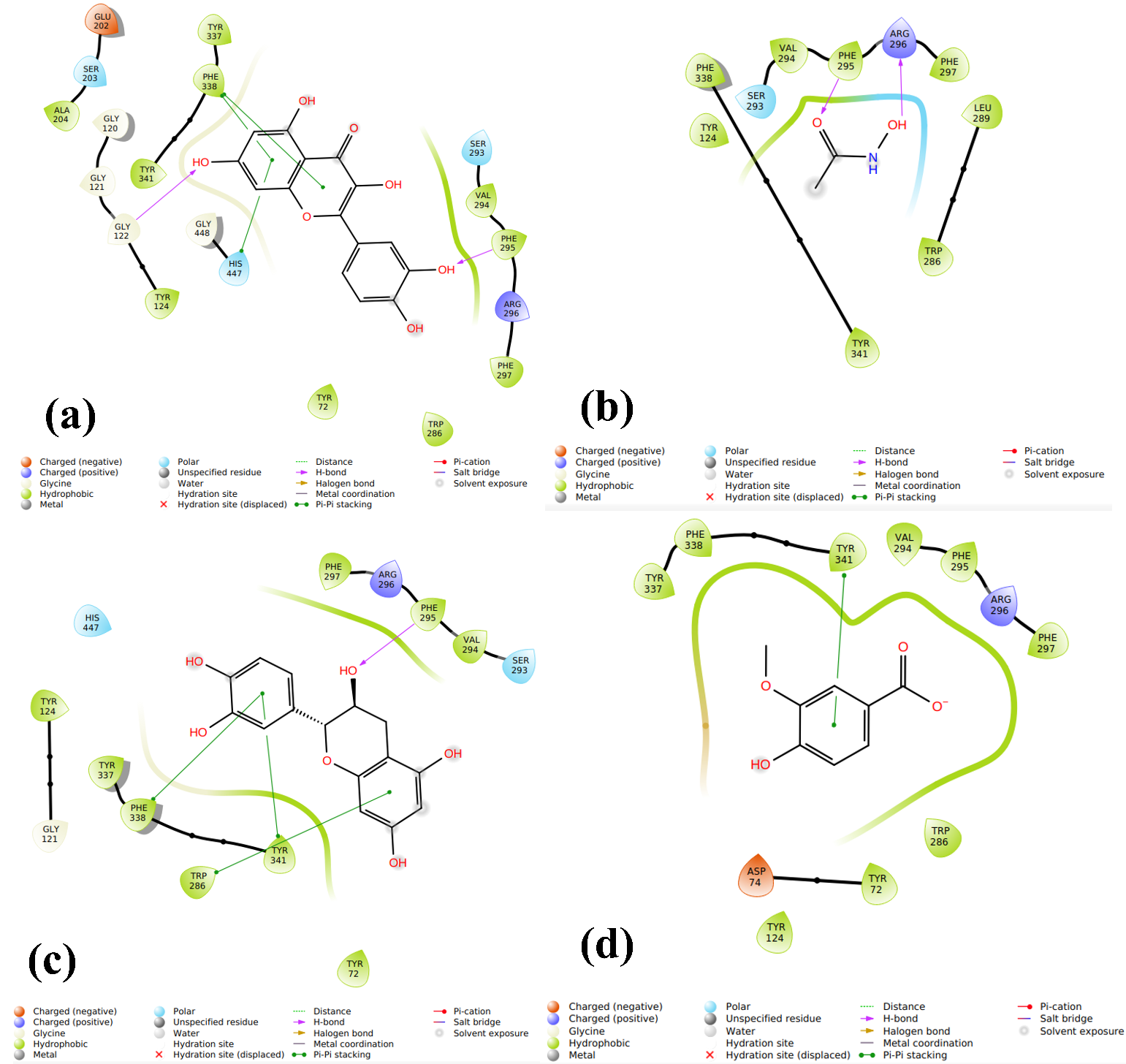
Figure S3.** The intricate atomic interactions between the ligands (a-t: MG1-MG20) and the essential amino acid residues at the active site of Recombinant Human Acetylcholinesterase (PDB ID: 4EY7) are thoroughly delineated


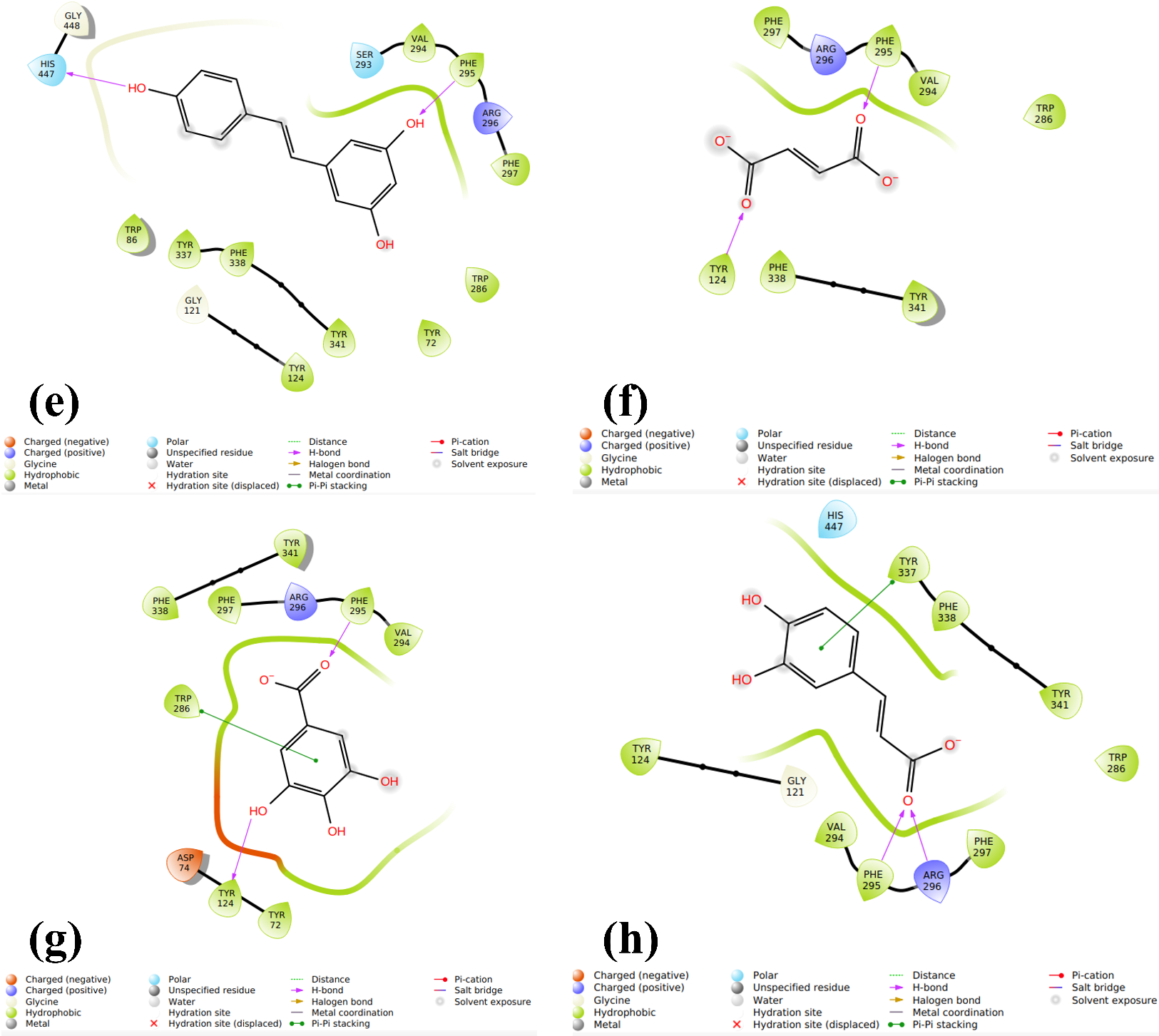


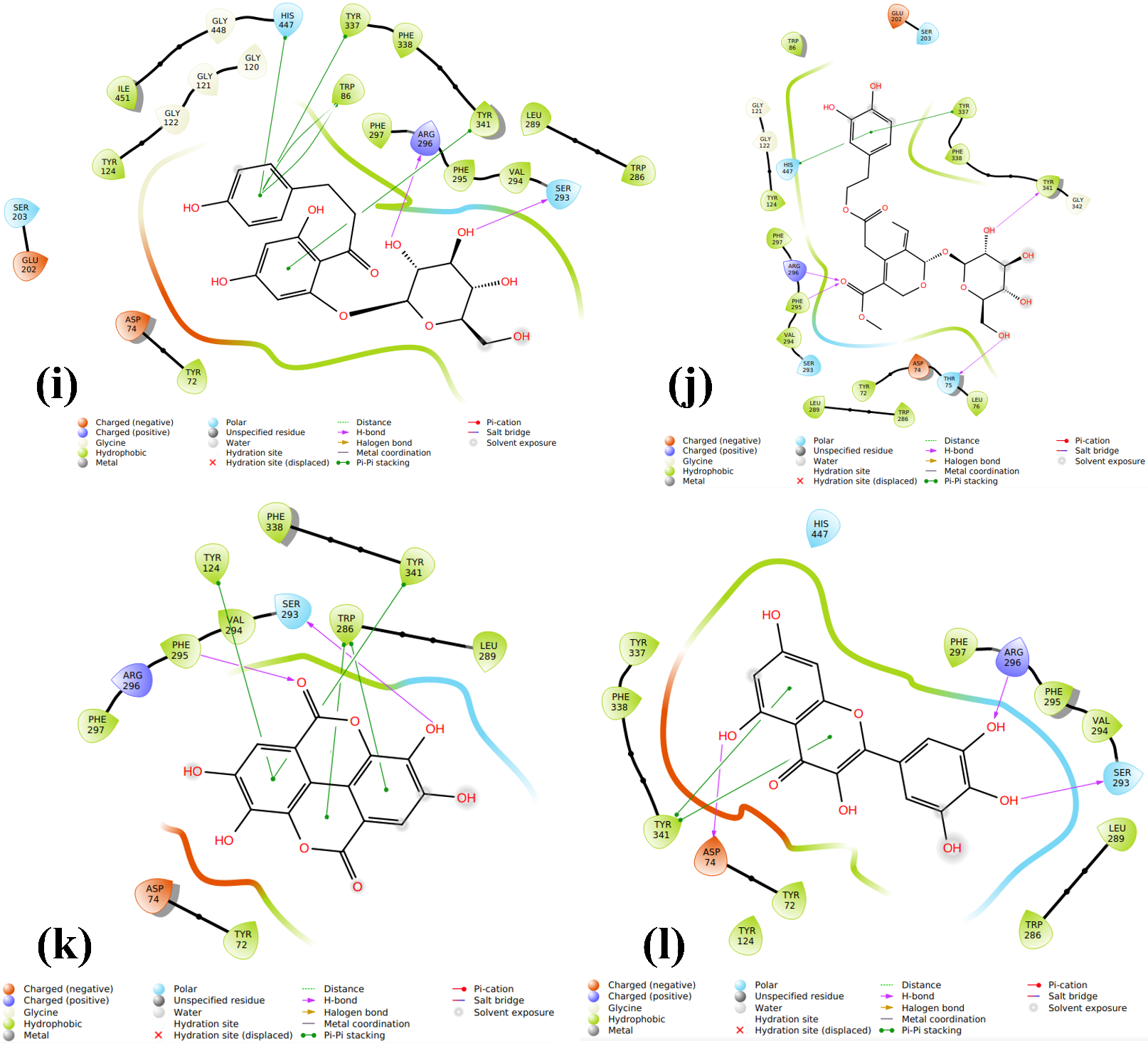


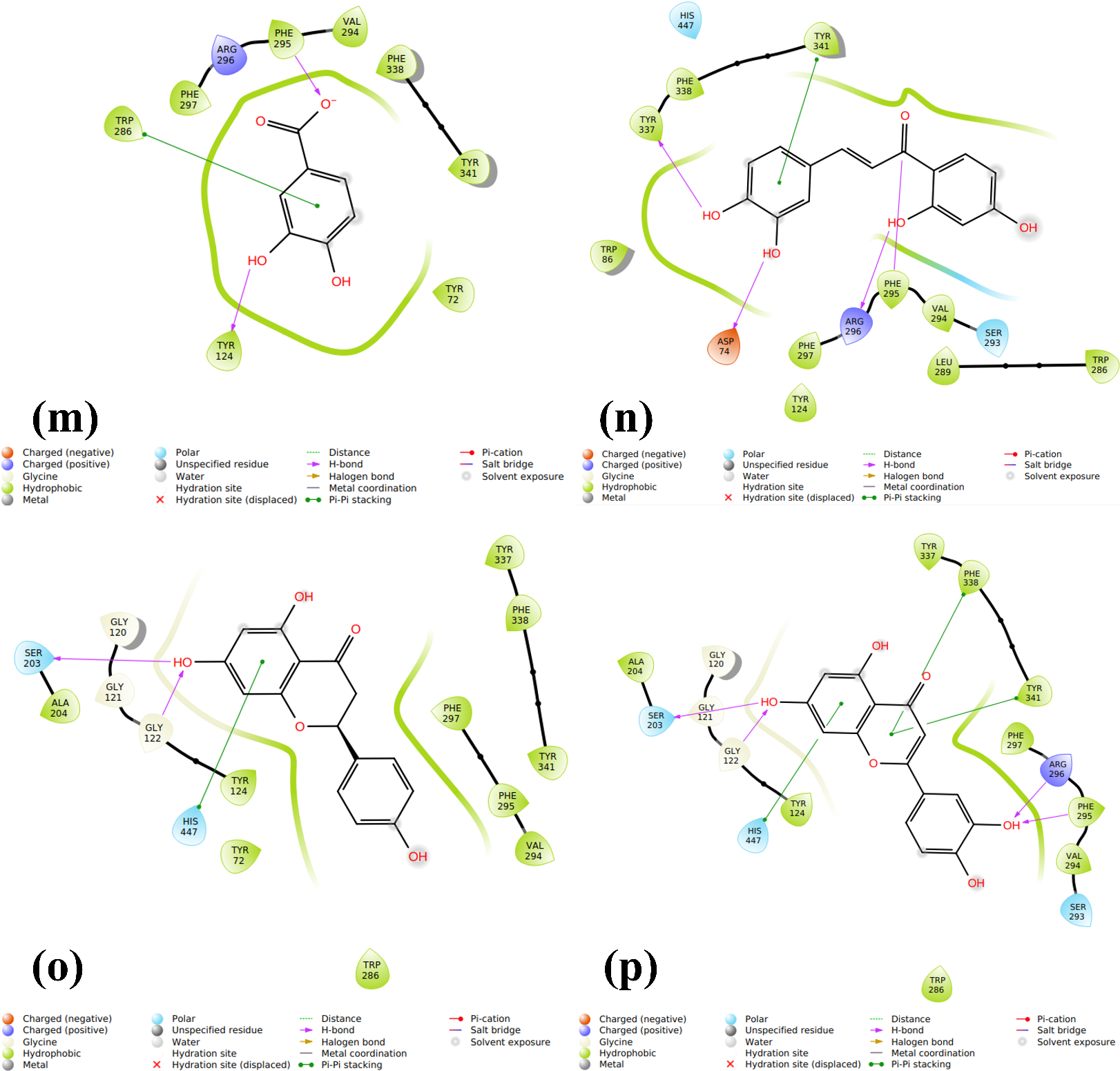


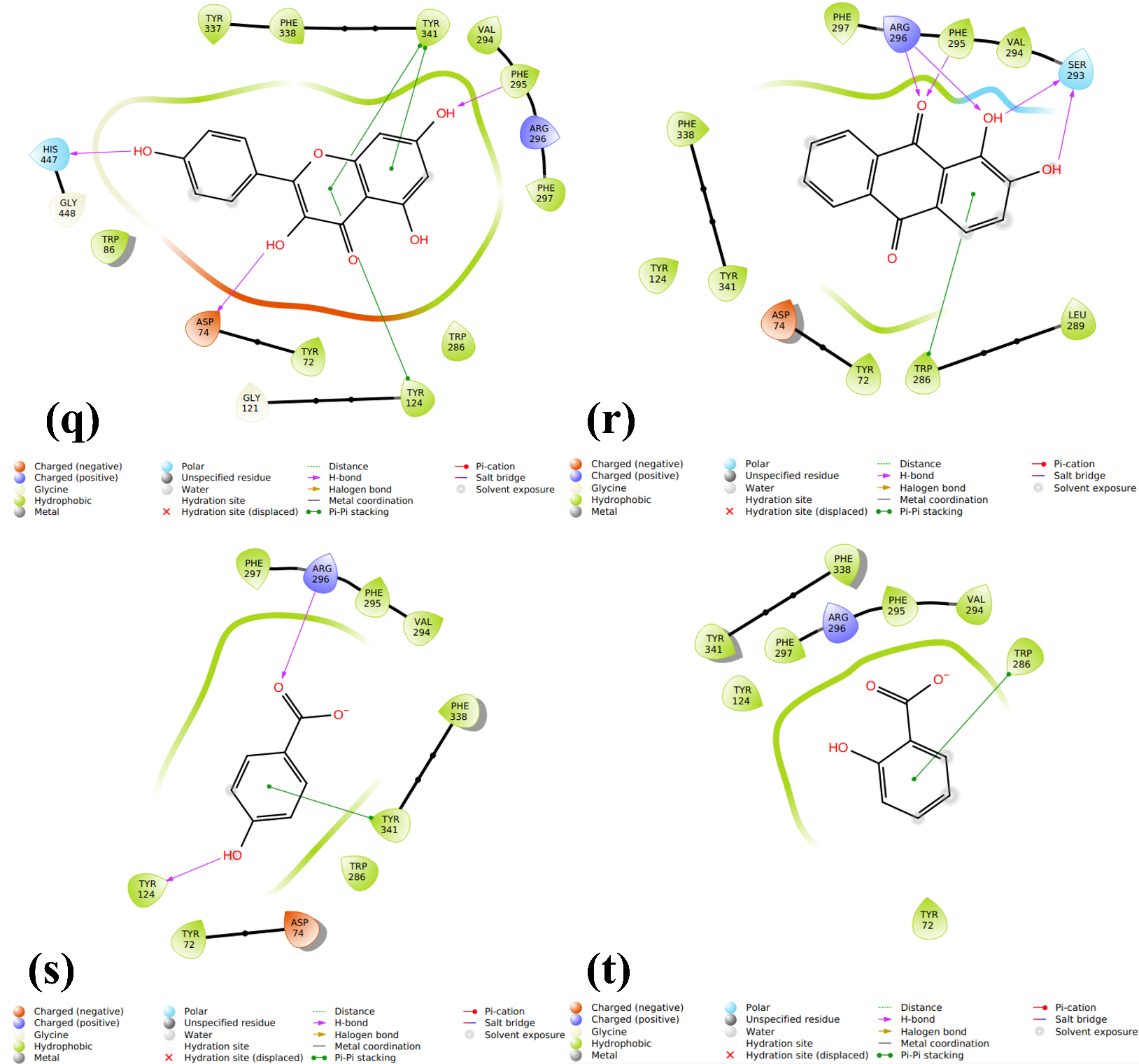


**Figure S4.** The intricate atomic interactions between the ligands (a-t: MG1-MG20) and the essential amino acid residues at the active site of Human butyrylcholinesterase (PDB ID:4TPK) are thoroughly delineated.


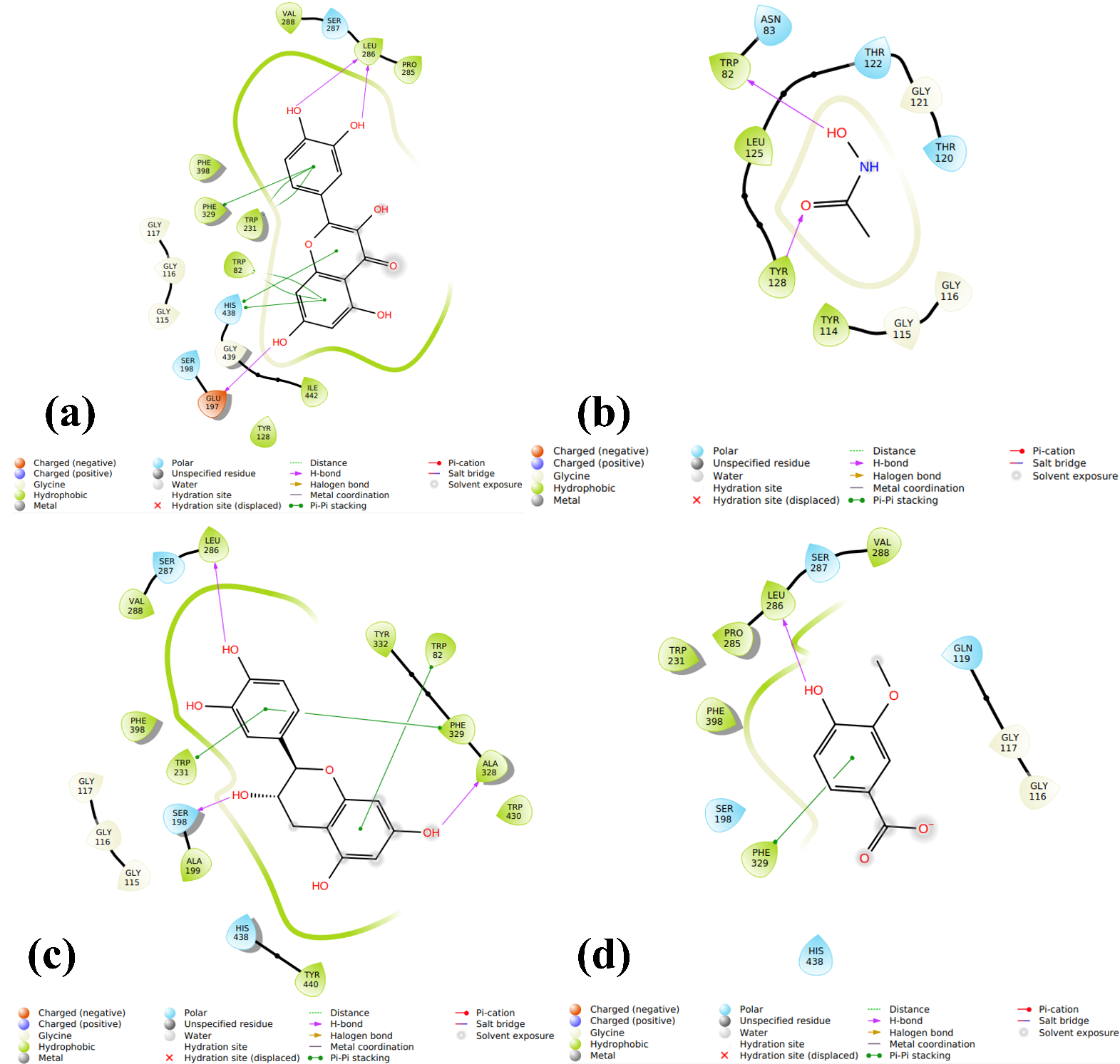


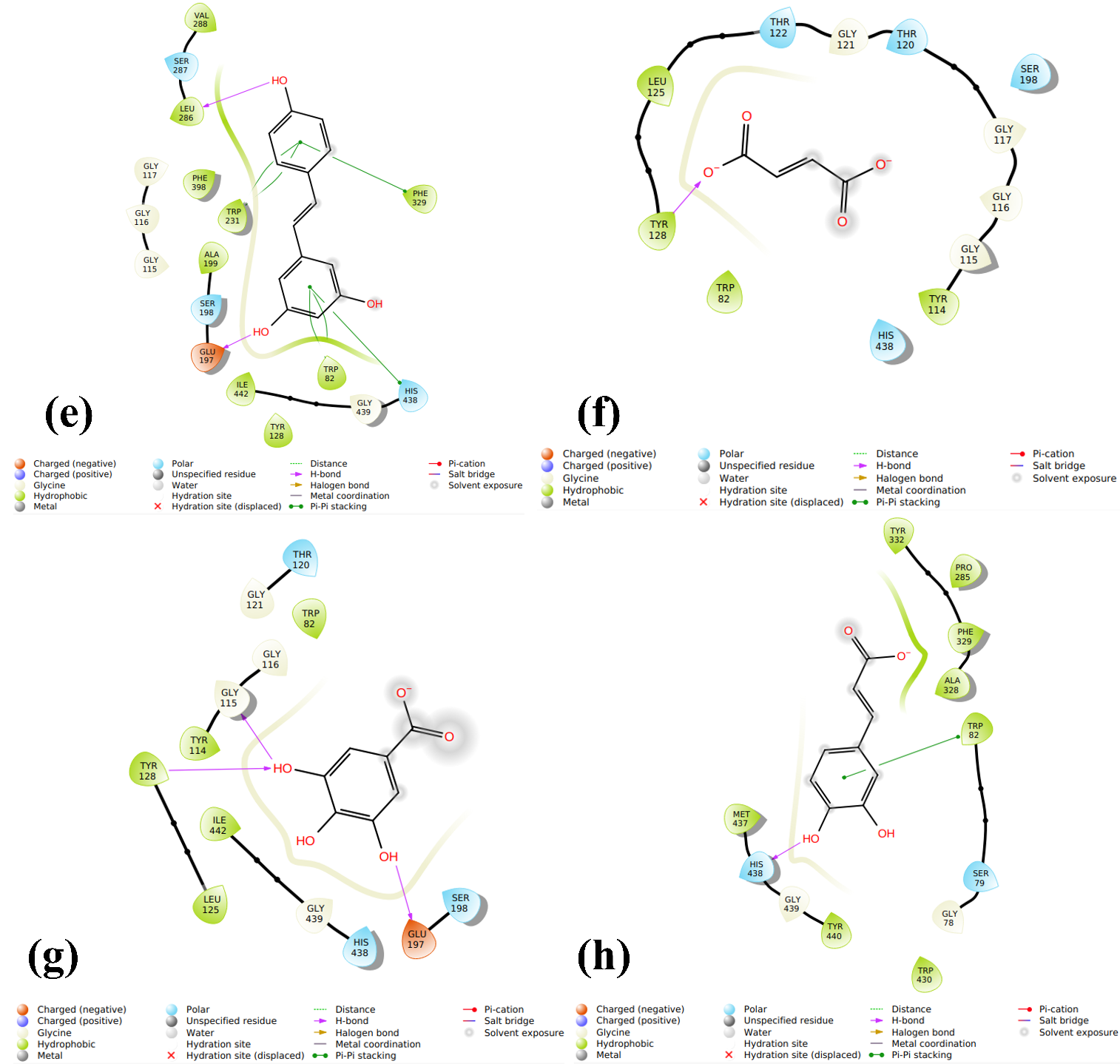


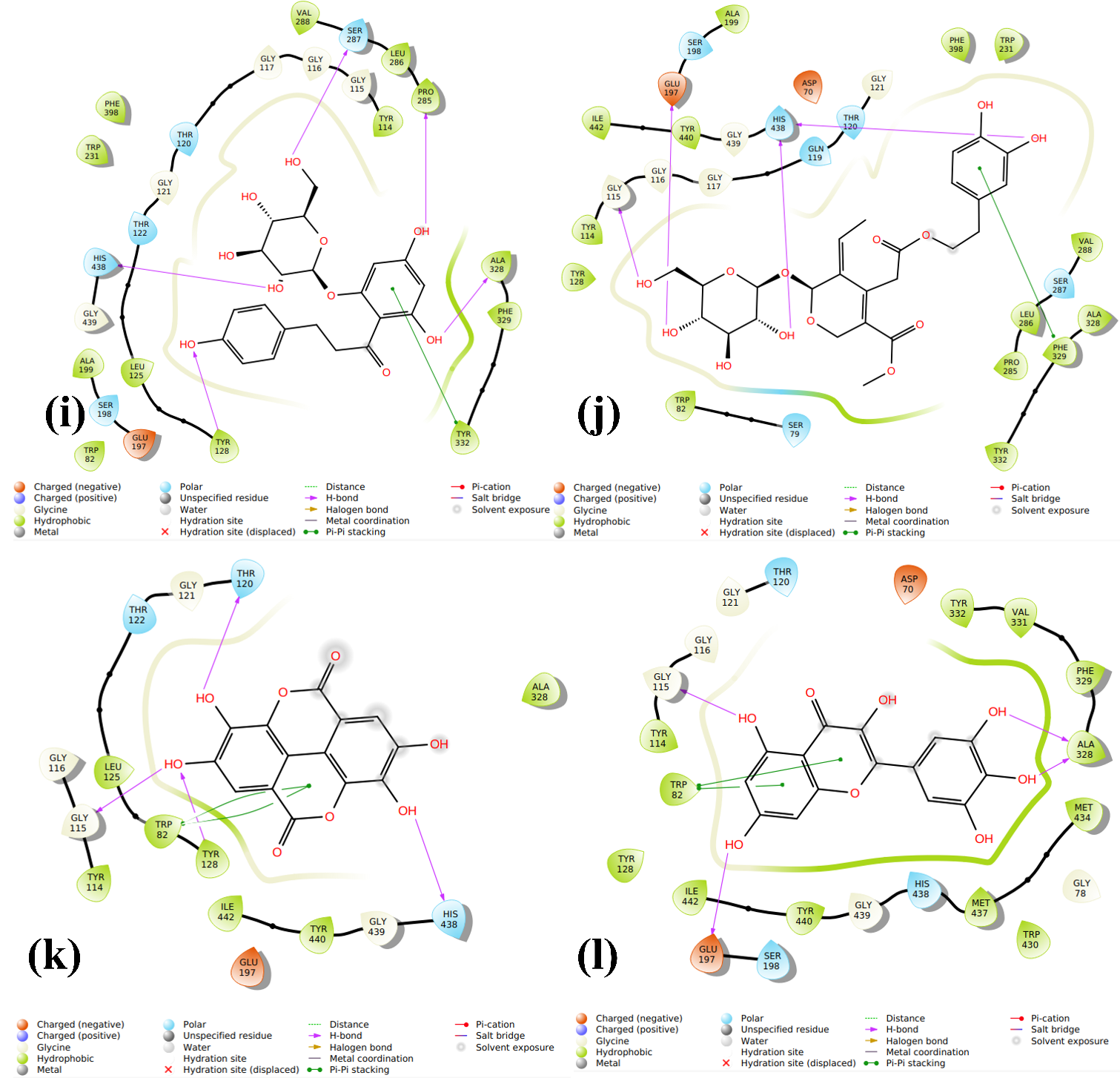


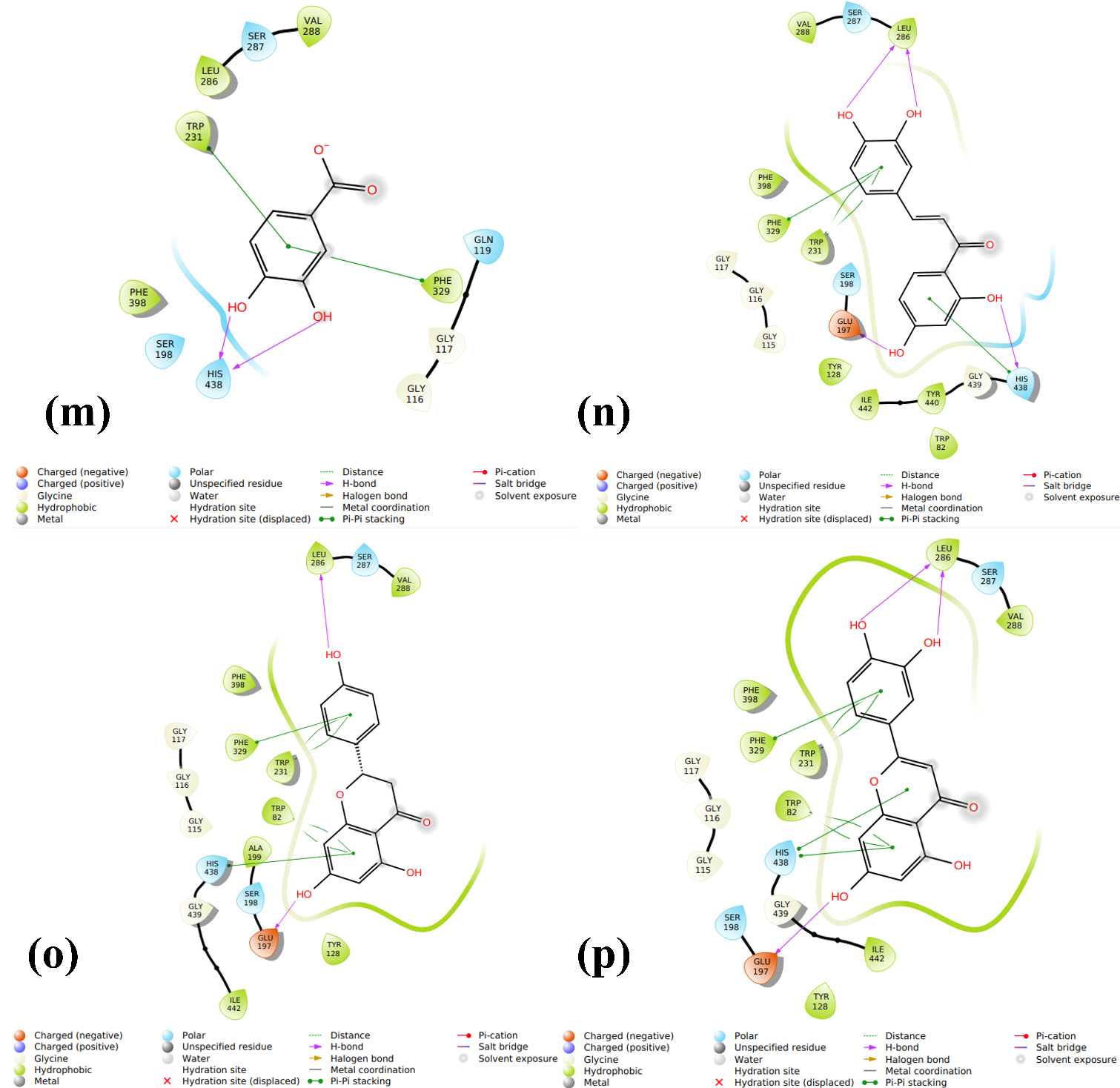


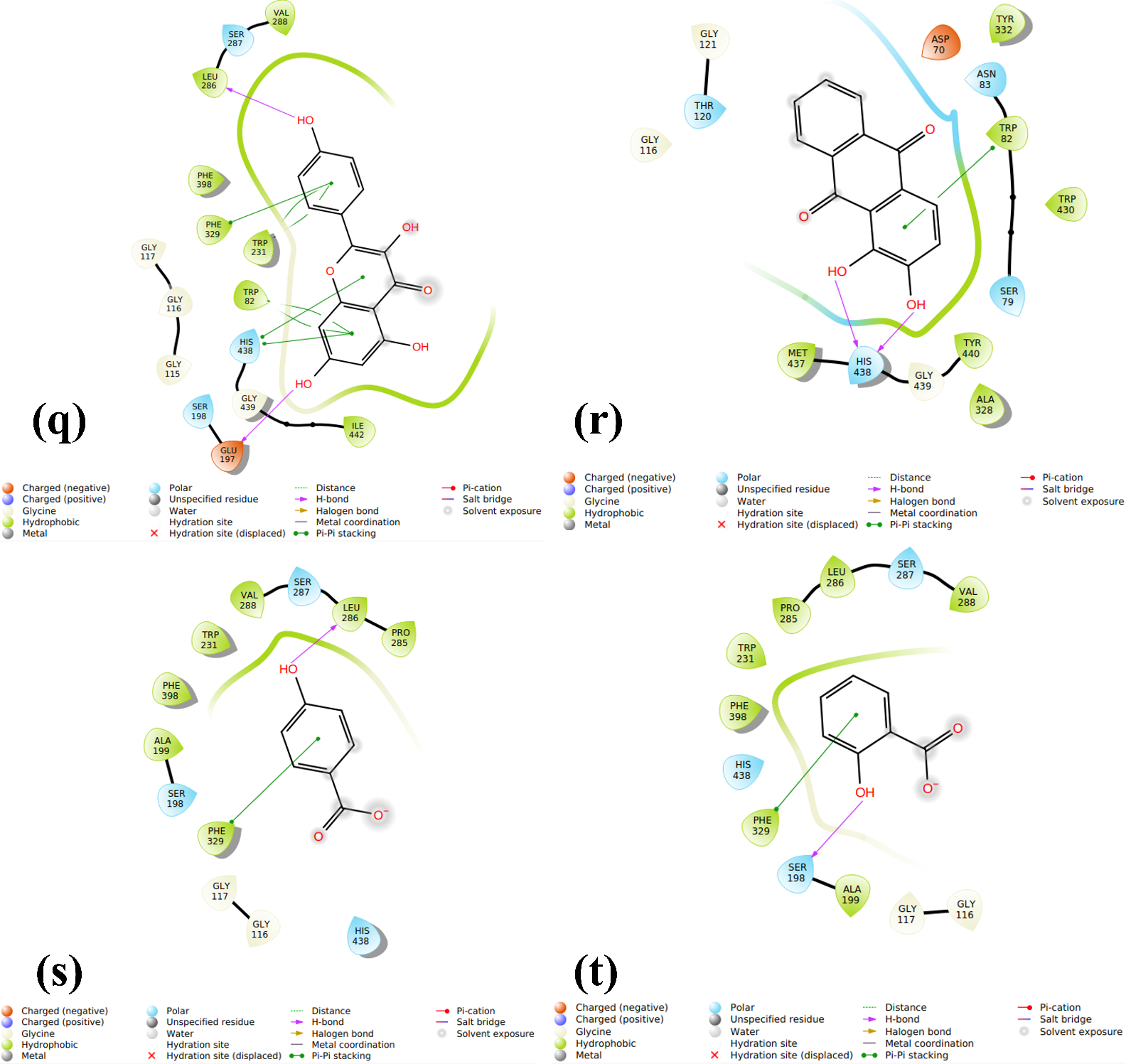


**Figure S5.** The intricate atomic interactions between the ligands (a-t: MG1-MG20) and the essential amino acid residues at the active site of Human lysosomal acid-alpha-glucosidase (PDB ID: 5NN8) are thoroughly delineated.


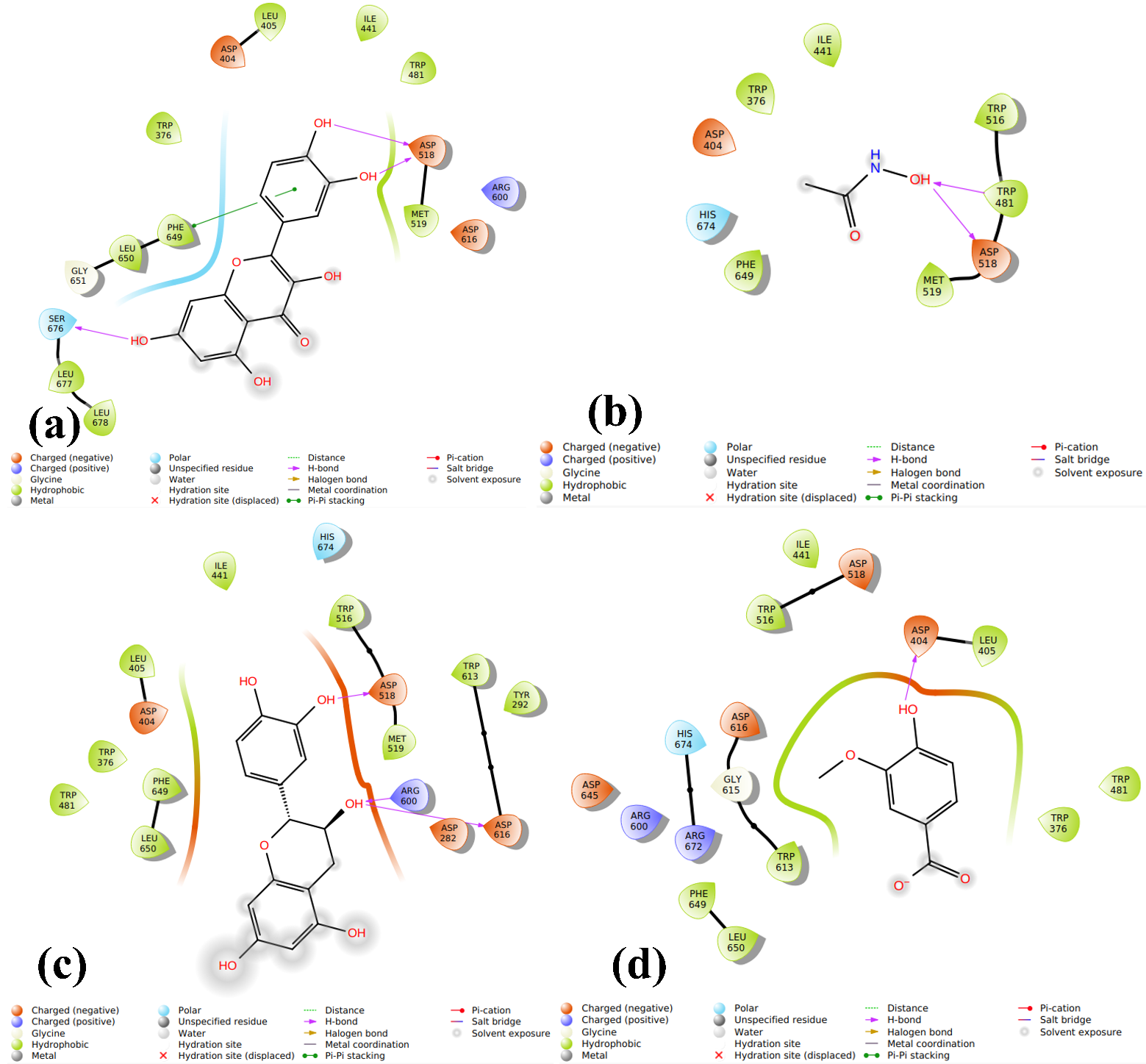


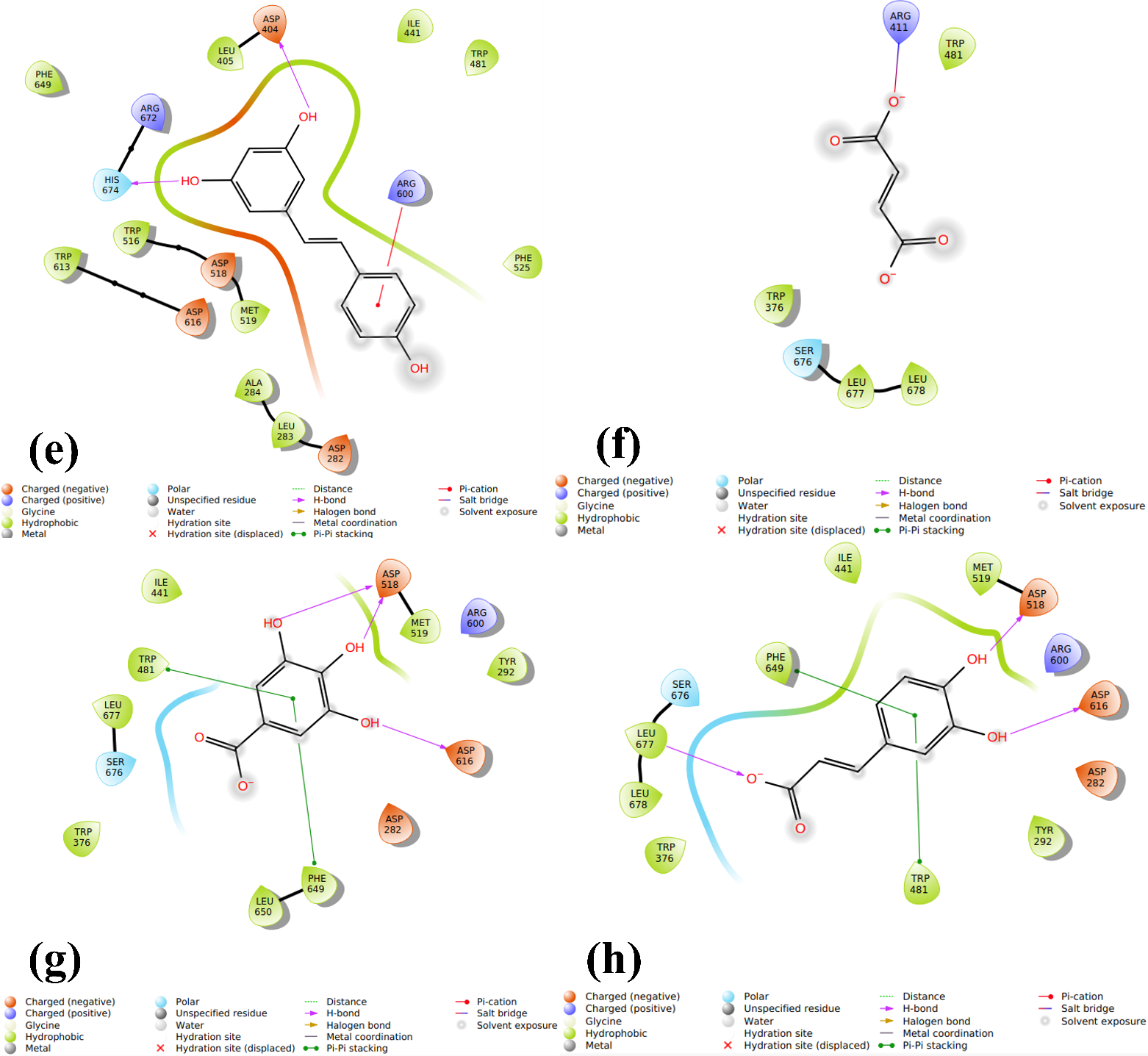


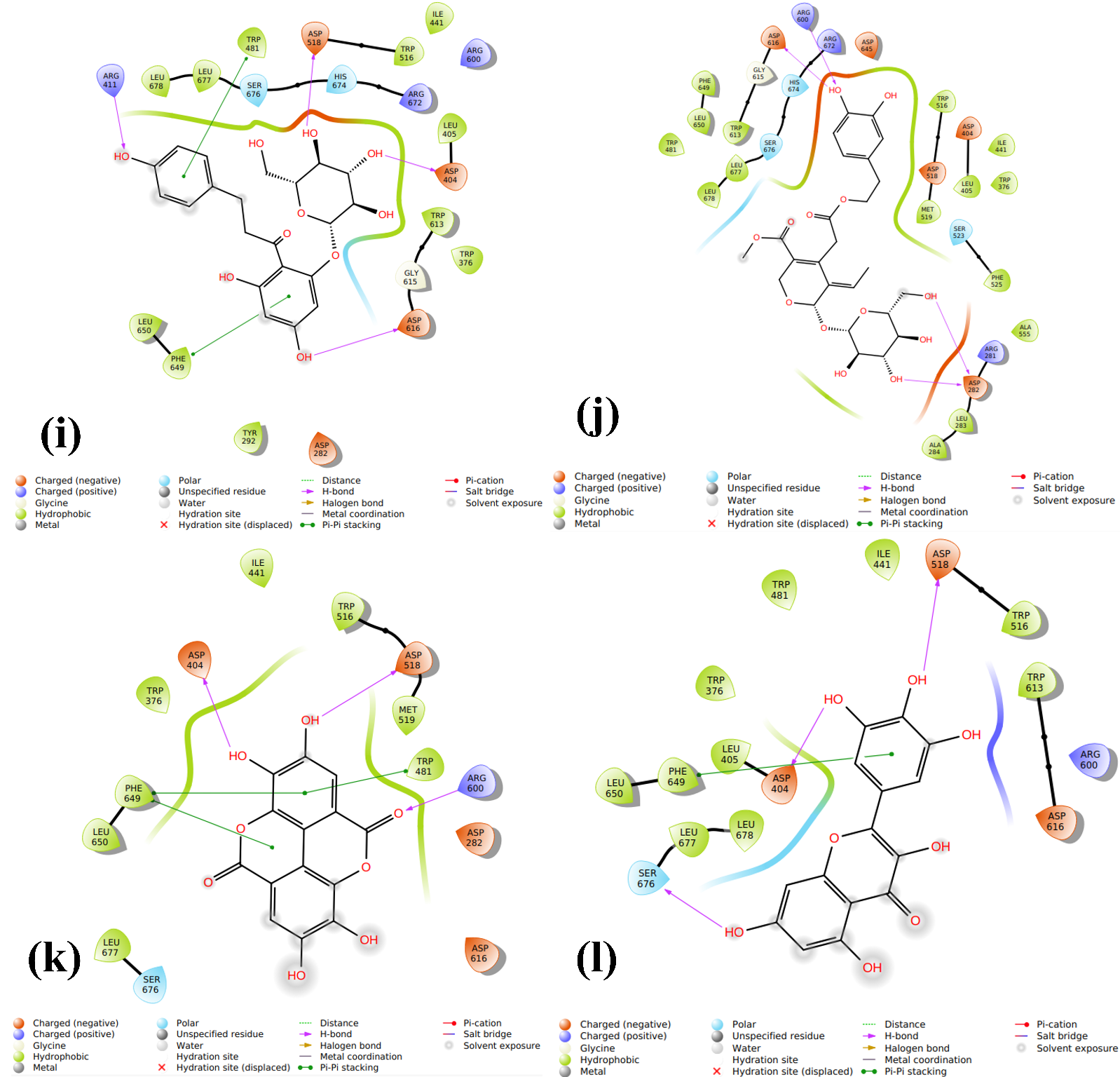


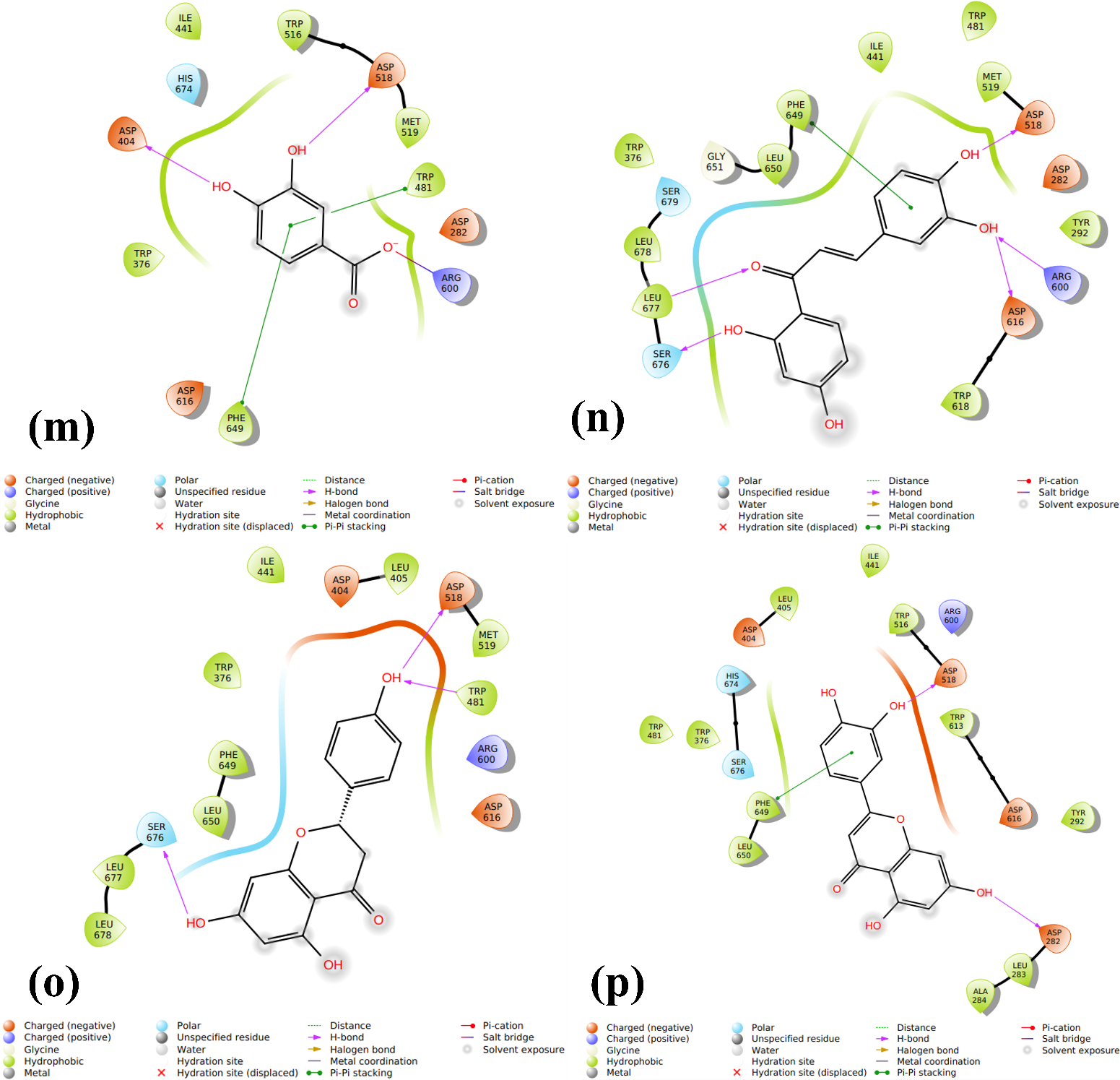


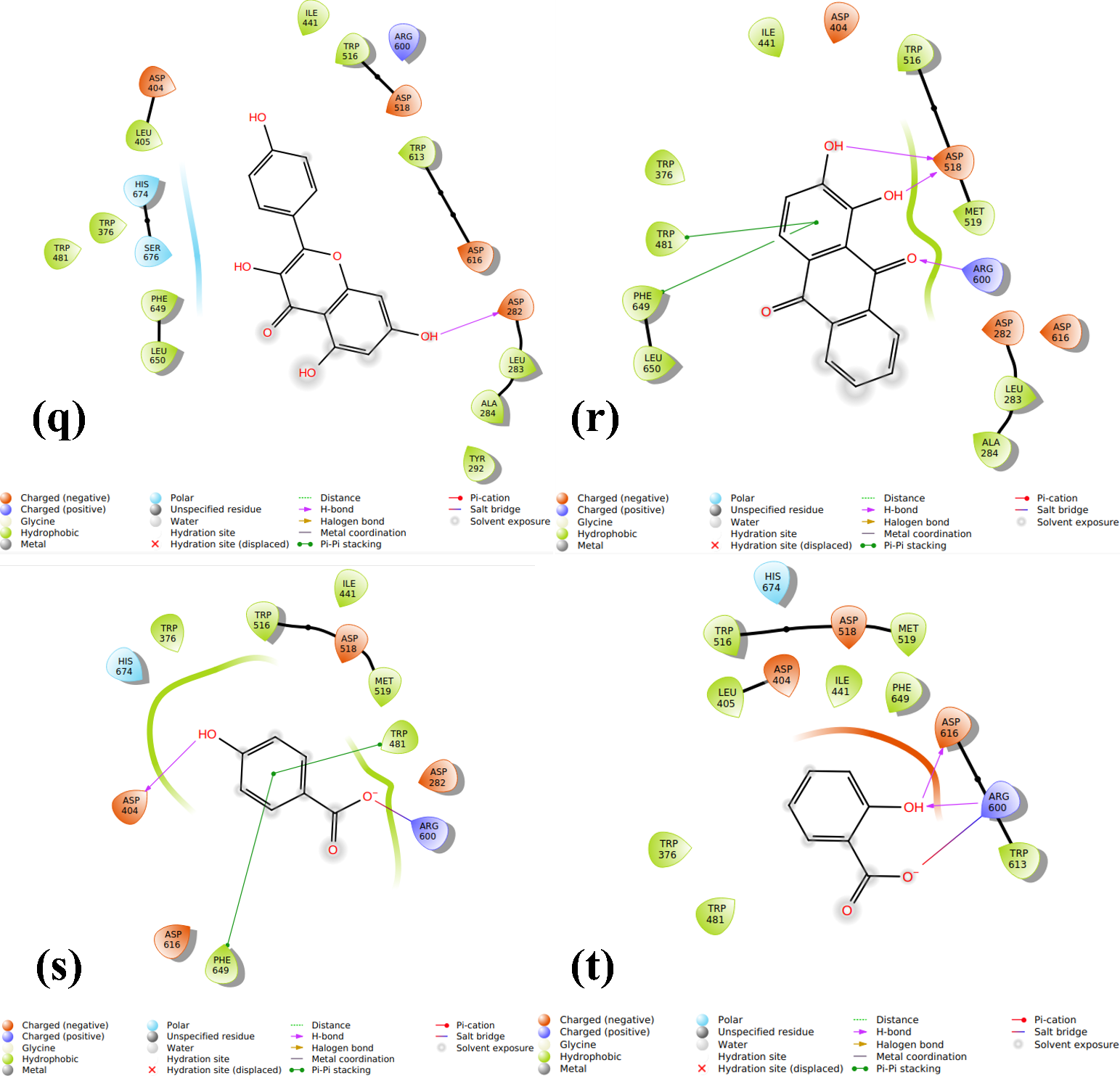

Supplement: Supplementary file 1 [file DataSheet1.docx]
